# Squash Under Strain: A Systematic Review and Meta-Analysis of Injuries and Illnesses in Squash Players

**DOI:** 10.3390/sports14020079

**Published:** 2026-02-11

**Authors:** Rachel Victoria McCartney, Thomas Fallon, Neil Heron

**Affiliations:** 1 School of Medicine, Dentistry and Biomedical Sciences, Whitla Medical Building, 97 Lisburn Road, Belfast BT9 7BL, UK; 2 Centre for Public Health, Institute of Clinical Sciences, Royal Victoria Hospital, Belfast BT12 6BA, UK; tfallon02@qub.ac.uk (T.F.); n.heron@qub.ac.uk (N.H.); 3 Edinburgh Sports Medicine Research Network & UK Collaborating Centre on Injury and Illness Prevention in Sport (UKCCIIS), Institute for Sport, PE and Health Sciences, University of Edinburgh, Edinburgh EH8 9YL, UK

**Keywords:** epidemiology, heat strain, illness, injury prevention, meta-analysis, racquet sports, sports injuries, systematic review

## Abstract

**Background:** Squash, a high-intensity sport with growing global popularity and an upcoming 2028 Olympic debut, is known to pose a wide range of potential health risks. However, epidemiological research of squash-related injuries and illnesses lacks consistency regarding reporting metrics and methodological standardisation. Therefore, this study aimed to systematically review the global literature to identify the incidence, prevalence, and anatomical distribution of reported squash-related health issues, calculate a pooled injury rate, and highlight research gaps. **Methods:** Following PRISMA guidelines (PROSPERO ID: CRD420251081709), a search conducted across MEDLINE, Embase, and Web of Science (from inception to 12 June 2025) yielded 12 studies, and a random-effects model estimated the pooled injury rate. **Results:** The pooled injury rate approximated 0.74 injuries per 365 athlete-days (95% CI: 0.26–2.07) and 2.01 injuries per 1000 athlete-days (95% CI: 0.72–5.67); however, extremely high heterogeneity (I^2^ = 99.65%) revealed significant methodological inconsistencies. Lower limb soft tissue injuries were most common, though regional patterns varied substantially. Additionally, risks from cardiovascular strain and hyperthermia were noted within the literature, alongside a generally poor uptake of protective equipment and a significant research gap on squash-related illnesses. **Conclusions:** Lack of standardisation hinders risk assessment and prevention within squash; therefore, future research requires an international consensus on injury surveillance, particularly as squash enters its Olympic era.

## 1. Introduction

### 1.1. Squash Overview

Squash has been hailed as one of the world’s most fast-paced, dynamic, and demanding sports [1,2,3,4] and it has become increasingly popular in today’s sporting world [1,5,6]. With over 15 million squash players spanning 135 countries [7,8,9], in addition to its upcoming debut within the Los Angeles 2028 Olympic Games [10,11], one can begin to appreciate its rapid growth of global popularity.

In squash, two players (or two teams of two) compete within a small court [5,7], using a racket to forcefully strike a rubber ball off the walls at high speeds (up to 230 km/h) to score points against their opponent [1,12,13]. Players can spend up to 70% of a match in active play [14], rapidly using energy through both aerobic and anaerobic pathways [15]. Therefore, prolonged rallies require significant muscular strength, endurance, and control [1,15] to enhance a player’s rapid accelerations and decelerations throughout constant lunging, side-stepping, and sprinting [2,8,11].

Rapidly unpredictable adjustments of dynamic movements, particularly velocity and direction, can yield a high-intensity activity with little recovery time [10]. Similarly, rapid and precise footwork can place substantial levels of stress onto lower limbs and joints and produce acute soft tissue-related or chronic overuse injuries across all player categories [2,8,15]. The upper limb may be negatively affected by repetitive shoulder swinging [4,16] due to repeatedly smashing the ball with a racket.

While the exact demands of squash take a variety of factors into consideration, such as skill level, match duration, and environmental conditions [15], it is generally accepted that higher fitness levels produce higher game quality [4,15,17]. Therefore, obtaining and maintaining higher fitness levels could improve a player’s performance, reduce fatigue, and decrease their relative injury risk [1]. Hence, as suggested by Verow [18] (pp. 876, 878), one “should be fit to play squash, not play squash to get fit”; the accuracy of this statement will be scrutinised deeper within this review.

### 1.2. Squash Injuries and Illnesses

Common squash-related injury sites include eye sockets, musculoskeletal (MSK) systems, and dental regions [2,7,8,13,19,20]. Whilst many eye injuries may be avoided through use of approved protective gear, there is generally a low uptake among players [21].

Acute and chronic MSK injuries are relatively common [2]. Whilst squash-related dental injuries were classified as “medium risk” by Persic, Pohl and Filippi [20] (pp. 231, 234–235), many players and coaches were not prepared to handle on-court dental emergencies [20]. Additionally, severe anomalous incidents following direct impact trauma, such as a vertebral artery dissection [22] and vertebral artery traumatic aneurysm [23,24], have been reported in the literature.

Moreover, significant thermal loads and heat strain can force a player’s heart rate and core temperature to dangerously increase up to 92% and 86% of their maximum, respectively, which could place an excessive, and potentially fatal, load on the cardiovascular system, restricting agility, balance, and coordination [6,10,25]. Furthermore, heat-related sickness, often associated with competitive indoor squash and exacerbated by dehydration, can contribute towards transient deterioration of cognitive function [6,10], limiting decision-making ability, footwork precision, and shot accuracy [10].

### 1.3. Surveillance of Sport-Related Injuries

A comprehensive review of global squash injuries and illnesses could provide an estimated relative incidence while addressing the root causes [3,15,26,27]. Consequently, squash injury and illness surveillance can quantify health risks, inform prevention strategies, influence global policies, and assess the impact of implemented safety measures [10,15,19,26,27], securing a progressively safer squash environment for players and coaches [1,2,10,19].

### 1.4. Gaps in the Literature and Study Rationale

Many studies have focused on the incidence and severity of squash injuries and illnesses; however, there is significant inconsistency regarding the definition of “injury” [12], which could lead to inaccurate reporting across the literature [3,27]. Moreover, there are presently no studies that collate squash injuries and illnesses within one review, subsequently evidenced and supported through systematic analysis of the literature. Additionally, studies often adopt contrasting methodologies and avoid comparisons between various demographics [3,12,16,17]. There are also few studies which accurately record exposure time across training and matches to calculate rates of injury or illness, limiting cross-study comparison [12]. Overall, these factors highlight the need for re-contemplation of classification standards within squash, especially in light of its growing popularity across the globe [5,7,11,13,15,16,28].

### 1.5. Study Aims and Objectives

The aim of this study was to perform a systematic review and meta-analysis of injuries and illnesses acquired while playing squash. The main objective was to analyse and collate evidence within the literature regarding the incidence, prevalence, and anatomical distribution of squash-related injuries and illnesses, with no demographical limitations. The author also planned to evaluate the effectiveness of modern reporting metrics within squash to encourage future standardisation of health risks across all sporting contexts.

## 2. Materials and Methods

### 2.1. Study Protocol

**Figure 1:**
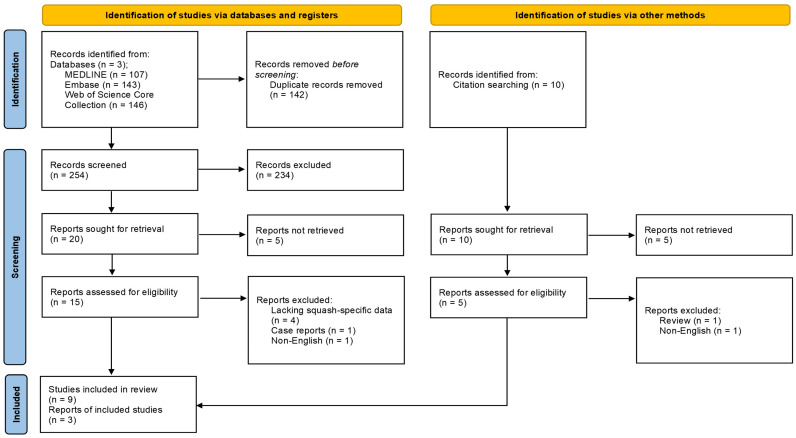
A PRISMA flow diagram illustrating the study selection process for squash-related injury and illness literature, outlining the identification, screening, and inclusion phases alongside reasons for exclusion.

This systematic review and meta-analysis followed the Preferred Reporting Items for Systematic Reviews and Meta-Analyses (PRISMA) guidelines, as detailed within Figure 1. The research protocol was submitted to PROSPERO on 20 July 2025 and published on 15 August 2025 (registration ID: CRD420251081709; available at https://www.crd.york.ac.uk/PROSPERO/view/CRD420251081709, accessed on 15 August 2025), as shown in Supplemental Material S1. Ethics approval was not required for this study.

The SPIDER (sample, phenomenon of interest, design, evaluation, research type) framework [29] was applied during the initial stages of the research process: squash players across all levels and demographics (sample); types of injuries and illnesses acquired by playing squash (phenomenon of interest); primary studies in English, excluding review articles, case reports, and non-original research (design); injury and/or illness incidence, prevalence, symptoms, and performance impact (evaluation); and mixed methods (research type).

### 2.2. Study Selection and Data Extraction

A comprehensive database search was conducted across MEDLINE, Embase, and Web of Science Core Collection from database inception to 12 June 2025: full search strings are detailed within Supplemental Material S2; reference lists were also screened. As MEDLINE constitutes the majority of PubMed content, the authors did not conduct a separate PubMed search to prevent excessive duplication of results. An updated database search was performed on 12 June 2025. Initially, 369 articles were retrieved across the three databases. Following manual de-duplication of studies, titles and abstracts were screened by two independent reviewers; discrepancies (n = 69) were resolved by discussion. Relevant data was extracted from included studies (n = 12) using a self-created tool (see Supplemental Material S3).

### 2.3. Methodological Quality Assessment

Study quality was assessed using the National Heart, Lung, and Blood Institute (NHBLI) Quality Assessment Tool for Observational Cohort and Cross-Sectional Studies [30]. The author calculated the total number of positive responses for each study, where a higher score suggested a lower risk of bias; see Supplemental Material S4 for full details.

### 2.4. Certainty of Evidence Assessment

The overall certainty of evidence was assessed using the Grading of Recommendations Assessment, Development and Evaluation (GRADE) framework [31] that labels evidence with a high, moderate, low, or very low level of certainty. Downgrading criteria included risk of bias, inconsistency, indirectness, imprecision, and publication bias.

### 2.5. Statistical Analysis

A random-effects meta-analysis was performed to calculate a pooled injury rate. Studies detailing the (1) number of squash athletes, (2) number of squash-related injuries, and (3) length of study in days were included (n = 8). All analyses were performed using R Statistical Software (v4.1.2; R Core Team 2021) applying the Sidik-Jonkman estimator and Hartung–Knapp adjustment; heterogeneity was calculated using the I^2^ statistic.

### 2.6. Equality, Diversity, and Inclusion Statement

This study included squash players of all genders, socio-economic backgrounds, and global populations. The author group included a medical student (RM), a medical doctor (NH), and a physiotherapist researcher (TF), all of whom reside in the UK.

## 3. Results

### 3.1. Overall Study Findings

A database search was performed as per PRISMA guidelines. Specific injury definitions and measurement units are expressed per study in Table 1, and data detailing the studies’ length, population size, and number of squash-related injuries is presented within Table 2. See Supplemental Material S5 for a comparison between study characteristics and Supplemental Material S6 for all major findings and risk factors reported.

### 3.2. Squash-Related Injuries

Meyer et al. [33] reported that 29% of participants sustained an injury within four weeks, producing an incidence rate of 0.45 injuries per 1000 h of exposure. However, Okhovatian and Ezatolahi [17] reported that 41 out of 52 (78.8%) experienced at least one injury over a two-year recall period, Sankaravel, Lee, Mondam and Low [3] stated that 83.33% of young Malaysian players reported MSK injuries over a 12-month recall period, and Jhamb and Singh [13] found that 86% of Indian club-level players had previously sustained an injury requiring more than two weeks to recover.

#### 3.2.1. Rates of Injury Incidence

Squash had the highest rate of injury incidence among youth multisport athletes, approximating 8.5 injuries per 1000 h of exposure over a five-year period [32]. This rate was significantly higher than other sports included within the same study and cohort, including gymnastics, table tennis, fencing, and track and field; notably, squash demonstrated a relative risk of injury over double that of fencing (approximating 3.99 injuries per 1000 h), highlighting the unique biomechanical and physiological demands placed on the squash player. Furthermore, Horsley, O’Donnell and Leeder [12] reported a mean of 8.83 injuries per professional player over an 11-year period. Whilst only a small percentage of squash injuries and illnesses may require hospital treatment, Eime, Zazryn and Finch [19] calculated the overall rate of hospital-treated squash injuries as 80.9 injured players per 100,000 players in Victoria, Australia, highlighting the importance of investigating the full spectrum of injury severity. Rejeb, Johnson, Vaeyens, Horobeanu, Farooq and Witvrouw [32] and Meyer, Van Niekerk, Prinsloo, Steenkamp and Louw [33] used standardised exposure-based metrics to report their findings as 8.5 and 0.45 injuries per 1000 h, respectively. However, Parkkari et al. [34] suggested that a player’s injury risk ranged from 6.6 to 18.3 injuries per 1000 h, which was significantly higher than low-risk activities such as walking, dancing, or swimming, which ranged from 0.19 to 1.5 injuries per 1000 h.

#### 3.2.2. Types and Mechanisms of Injuries

Reported squash injuries most commonly encompassed soft tissue damage, including strains, sprains, tendonitis, bursitis, acute trauma, and overuse. This category accounted for 71.11% of a cohort of elite professionals [12] and 85% of a cohort of Nigerian squash players [4]. Notably, MSK injuries were the most frequently reported injury type across most studies, spanning 44.5% of recreational players [36], 83.33% of young Malaysian squash players [3], 86% of Indian club-level squash players [13], and nearly nine injuries per professional over a period of 11 years [12]. Muscle-related injuries (including strains, cramps, soreness, and trigger points) collectively formed 37.50% of all injuries within a study by Horsley et al. [12]. Over a third and half of reported cases within studies by Berson, Rolnick, Ramos and Thornton [36] and Eime, Zazryn and Finch [19], respectively, were labelled as strains and sprains; tendonitis was most frequent (22.64%) amongst professional English players [12].

Chard and Lachmann [35] and Talabi, Olaitan, Bakinde and Onigbinde [4] reported that 80% and 52.5% of injuries within their populations were acute, respectively. However, Rejeb, Johnson, Vaeyens, Horobeanu, Farooq and Witvrouw [32] suggested that 50.3% of young athletic injuries within their population were due to overuse, since overuse and acute injuries were calculated as 4.7 and 3.3 per 1000 h of exposure, respectively. Talabi, Olaitan, Bakinde and Onigbinde [4] and Meyer, Van Niekerk, Prinsloo, Steenkamp and Louw [33] reported a similar trend, where 18.2% of Nigerian players and 42% of South African adolescents sustained an overuse injury, respectively.

Persic, Pohl and Filippi [20] (pp. 231, 234–235) described squash-related dental trauma as “medium risk”, often caused by collisions with equipment or an opponent. It was reported that 4.5% of their population had personally sustained and 20.4% had observed a squash-related dental injury. Furthermore, 20 out of 27 participants had experienced a crown fracture while 89 out of 142 had witnessed one [20]. Despite these known risks, only 1 out of 600 participants reported that they regularly wore a mouthguard during play [20], highlighting a gap in applied protective practices.

Eye injuries were less commonly reported across the literature: Eime, Zazryn and Finch [19] estimated an injury rate of 19.0 per 100,000 players, and Jhamb and Singh [13] calculated an overall incidence of 0.96%. However, these cases represented a significant proportion of severe injuries, often following direct trauma by a ball, racket, or opponent [19,20]. Eye injuries accounted for almost a third of emergency department presentations in a study by Eime, Zazryn and Finch [19], where consequences ranged from mild to very severe. Despite this evidence, there was still a low uptake (10%) of protective eyewear reported among Iranian players [17].

##### Lower Limb Injuries

The lower extremity was most commonly injured in squash across many studies, accounting for 32% to 76.48% of all reported cases [3,4,12,13,19,33,35]. Specifically, this type spanned 67.0% of time-loss injuries amongst young athletes [32], 63% of Nigerian players [4], 67.3% of Indian club players [13], and 76.48% of English professionals [12]; Chard and Lachmann [35] further proposed that 58% of squash injuries impacted the lower limb.

Ankle and knee joints were noted as especially vulnerable: Finch and Eime [37] estimated a prevalence between 32% and 58%, Eime, Zazryn and Finch [19] proposed a prevalence of 68%, and Horsley, O’Donnell and Leeder [12] reported a prevalence of 76.35%. These joints were often associated with extended recovery times, averaging 9.25 months for combined knee injuries [13]. Among elite professionals, the ankle was found to be the most prevalent (20.81% of all injuries), followed by the thigh (12.69%) and knee (10.63%) [12]. Similarly, Jhamb and Singh [13] determined the knee joint to be most affected (21% of all injuries), followed by the ankle joint (8.3% of all injuries). Talabi, Olaitan, Bakinde and Onigbinde [4] recorded 30.2%, 20.5%, and 13.2% of all injuries affecting the ankle, knee, and foot, respectively, amongst a Nigerian cohort. Finally, in a Malaysian study by Sankaravel, Lee, Mondam and Low [3], ankle and knee injuries were the most frequently reported at 26.7% and 20% of all injuries, respectively. Female squash players commonly reported calf strains [35], whilst thigh injuries were the most reported injury (19%) amongst young squash players in the Western Cape, possibly influenced by growth-related imbalances between bone length and muscle flexibility [33].

##### Upper Limb Injuries

Upper limb injuries accounted for 19.23% [13], 15.5% [4], and 8.45% [12] of injuries across Indian, Nigerian, and English cohorts, respectively. However, there were discrepancies regarding the most affected joint. For example, 50% of elite players within a study by Horsley, O’Donnell and Leeder [12] and 13% of adolescent squash players in a separate study by Meyer, Van Niekerk, Prinsloo, Steenkamp and Louw [33] had sustained a shoulder injury, which was the most common injury type. Moreover, wrist and hand injuries were most prevalent (31.7%) in a Malaysian study of young players over a period of 12 months, followed by shoulder (25%) and elbow (8.3%) injuries [3]. In addition, both an Iranian and a Nigerian study described the elbow as the most injured upper extremity joint, accounting for 21% [17] and 6.2% [4] of total injuries, respectively.

##### Trunk and Spinal Injuries

Furthermore, many injuries concerning the spine and lower back regions have been reported. Lower back pain was particularly noted as the most common injury type amongst Iranian squash players at 36.5% of total injuries [17], where the authors suggested an influence of continual trunk movements in both rotational and sagittal planes. Lower back injuries accounted for 31.7% and 13% of total injuries in an Indian and a South African study, respectively [13,33]. Lastly, Horsley, O’Donnell and Leeder [12] concluded that spinal injuries in elite players, from most to least reported, included the lumbar (46.07%), cervical (25.84%), and thoracic (20.22%) regions.

#### 3.2.3. Injury-Related Risk Factors

Predominant squash-related risk factors included (1) older age, (2) high playing frequency, (3) inadequate warm-up, (4) limited skill, and (5) previous injury. In a study conducted by Jhamb and Singh [13], the mean age of participants was 41 years, and the mean age at the time of injury was 34 years; similarly, Chard and Lachmann [35] calculated 59% of the injured population to be over 25. However, whilst older players (particularly those over 40) may have been more vulnerable to injury and required longer recovery periods [36], young competitive players may also have experienced a high injury risk due to greater MSK demands [33]; increasing training time reportedly increased the relative risk (RR = 1.03 for every 10 h increase) of a young player sustaining an overuse injury [32]. Comparably, Parkkari, Kannus, Natri, Lapinleimu, Palvanen, Heiskanen, Vuori and Jarvinen [34] suggested that risk decreased with age, and Horsley, O’Donnell and Leeder [12] reported the highest rates of injury among younger professionals aged 18–23 years (41%).

Injury rates were generally inversely related to a player’s ability. Beginner players often showed limited spatial awareness, reduced racket skill, and incomplete knowledge of squash rules, and so were potentially more prone to self- and opponent-inflicted injuries [20,36]. Berson, Rolnick, Ramos and Thornton [36] further reported more injuries among beginner players, and Persic, Pohl and Filippi [20] noted more dental injuries across amateurs. However, Meyer, Van Niekerk, Prinsloo, Steenkamp and Louw [33] reported that elite players experienced a higher rate of injuries (41%) than those who played in school. Interestingly, Berson, Rolnick, Ramos and Thornton [36] suggested that whilst lower-level players may have sustained more superficial wounds, higher-level players often presented with more severe orthopaedic injuries requiring longer recovery periods.

Regarding the impact of gender, Eime, Zazryn and Finch [19] reported that male players accounted for 90% of all hospital admissions and nearly 80% of all emergency department presentations, and Chard and Lachmann [35] suggested a male-to-female injury ratio of 2:1. Whilst one study reported that female players suffered more injuries within endurance sports [34], and another that they sustained more injuries from ball-kicking (31%) [17], squash-related injuries were generally more prevalent among male athletes within both recreational and competitive environments [34].

Inadequate warm-up was often associated with higher injury incidence rates, regardless of skill level [35]. Meyer, Van Niekerk, Prinsloo, Steenkamp and Louw [33] proposed that warming up prepares the body for exercise and reduces injury risk by increasing blood flow to muscles, quickening nerve impulses, increasing range of motion, and loosening connective tissue. It was also suggested that adolescent squash players may sustain more injuries in the absence of suitable warm-up exercises (43%) compared to those who effectively warmed up (27%); the quantifiable impact of specific warm-up regimes falls outside the scope of this review. Additionally, previously injured players may be more vulnerable to recurrence [33,35].

### 3.3. Squash-Related Illnesses

#### 3.3.1. Cardiac Events and Mortality Risk

Whilst the literature provides extensive detail regarding squash-related injuries, there is limited data regarding squash-related illnesses. However, previous authors have discussed the significant cardiovascular strain which can follow competitive squash, where consequences can span cardiac injury, heat illness, and even death [38]. Additionally, pregnant women should be cautious when playing squash, as research has suggested that severe exercise-induced hyperthermia may induce congenital abnormalities in an unborn child [15].

A prospective study by Ihsan, Kwok, Wong, Girard and James [10] reported that a squash player’s heart rate may exceed 90% of its maximum during play, placing significant strain on the heart’s functional load and exacerbating the body’s thermal strain. The same study reported that muscle cramps, which are commonly experienced among elite players, may be an early warning sign of heat-related illness [10].

On the one hand, an epidemiological study by Finch and Eime [37] reported that, due to the intense levels of stress on the cardiovascular system, men over the age of 40 carried the highest risk of mortality across various sports, including squash. Furthermore, a retrospective examination of British media reports from 1983 to 1997 suggested that, out of the 30 cases of squash-related sudden deaths, 23 were linked with coronary heart disease [39]. On the other hand, whilst a 10-year-long study conducted by Quigley [40] in the Republic of Ireland reported 51 cases of sudden death within sport, only one of these was related to squash.

Moreover, a 1984 review investigating squash-related sudden deaths revealed that, within 60 cases spanning 8.5 years, the average age of mortality was 46 years old, where only one individual was female [39]. Coronary heart disease was the primary cause of 51 cases, equating to 85% of the sample population; interestingly, 75% of the cases had previously reported at least one symptom associated with cardiac disease within the final week of their life [39].

#### 3.3.2. Heat-Related Illnesses

Ihsan, Kwok, Wong, Girard and James [10] described squash as a technically and physically demanding sport that can cause players to cultivate substantial levels of metabolic heat production. This process may be exacerbated by high levels of play intensity and/or duration and the indoor playing environment, which can limit evaporative potential and cool-down opportunities [10]. Relevant risk factors included advancing age, low fitness level, obesity, and chronic conditions such as hypertension or diabetes [15]. Players can experience significant core temperature increases during matches; Ihsan, Kwok, Wong, Girard and James [10] suggested that elite players can reach core temperatures between 39.0 °C and 40.1 °C. Moreover, Nybo et al. [41] reported that core temperature increases above 38.5 °C may limit a player’s maximum muscle strength, endurance, and oxygen uptake.

Similarly, Ihsan, Kwok, Wong, Girard and James [10] suggested that significant hyperthermia can impair athletic performance, cognitive function, proprioception, and balance, which can compromise decision-making and technical ability [41,42,43]. Retrospective application of this knowledge to an older study by Blanksby et al. [44], where peak rectal temperature responses (n = 27) spanned 37.0 °C to 39.0 °C within 39 min of play, highlights the reality of acquiring severe squash-related illnesses in intense play.

### 3.4. Meta-Analysis of Pooled Results

**Figure 2:**
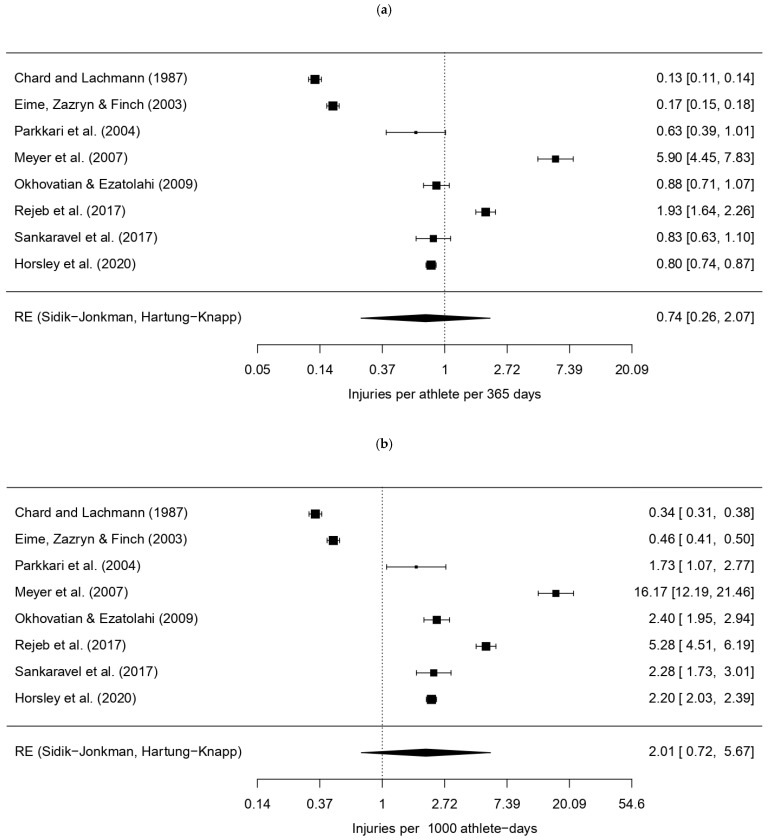
Forest plots displaying pooled injury incidence rates across studies [3,12,17,19,32,33,34,35], including confidence intervals, expressed per: (**a**) 365 athlete-days; (**b**) 1000 athlete-days.

Following manual extraction of reported injuries across all included studies, data was collated by anatomical region and tissue type within Supplemental Material S7, respectively. Confidence intervals (CIs) and proportional distributions were subsequently calculated to support comparative analysis of injury patterns. A random-effects model, combining data from eight studies (k = 8), calculated a pooled squash-related injury rate of approximately 0.74 injuries per 365 athlete-days (95% CI: 0.26–2.07) or 2.01 injuries per 1000 athlete-days (95% CI: 0.72–5.67), as shown by forest plots in Figure 2. These results were statistically significant (*p* < 0.0001), and applied units are explained within Table 3.

Regarding heterogeneity, the Q-statistic was highly significant, where Q(df = 7) = 1892.2864 (*p* < 0.0001), indicating substantial methodological variance across the included studies. A high I^2^ value of 99.65% supported this finding, suggesting that the high variability in results was due to true heterogeneity rather than random sampling error. The variance (τ^2^) between studies was estimated at 1.5149 when considering data expressed as injuries per 365 or 1000 athlete-days, further highlighting the significance of true heterogeneity.

Individual findings confirmed highly diverse incidence rates, ranging from 0.34 [35] to 16.17 injuries [33] per 1000 athlete-days. Nonetheless, lower limb injuries were most commonly reported, ranging from 48% [36] to 76.48% [12] of reported cases.

## 4. Discussion

### 4.1. Summary of Main Findings

Overall, MSK injuries were most reported across the literature. Most cases involved the lower extremity, specifically ankle and knee joints, although prevalence varied from 32% to 76.48% [3,4,33,35,36]. Other frequently reported regions included the lower back [3,4,13,17,33,35] and shoulder [3,12,33]. However, exact rates were inconsistent, possibly due to variations in training loads and recall periods. While eye and facial injuries were less common, they carried a higher severity risk [12,19,20,36]. Strains and sprains were frequently reported by Talabi, Olaitan, Bakinde and Onigbinde [4], Eime, Zazryn and Finch [19], Berson, Rolnick, Ramos and Thornton [36], Chard and Lachmann [35], and Horsley, O’Donnell and Leeder [12]. Moreover, three studies suggested that overuse injuries were increasingly common, especially among younger athletes [3,32,33].

Despite the recognised intensity of squash, it appeared to carry only a moderate risk of injury when compared to other sports. Individual studies reported squash-related injury rates from 0.45 to 18.3 injuries per 1000 h, which classified squash as lower risk than high-contact sports such as rugby (15 to 40 injuries per 1000 h) and football (6 to 9 injuries per 1000 h) and akin to or below the risk within tennis (1 to 3 injuries per 1000 h) and basketball (7 to 14 injuries per 1000 h) [34,37]. Furthermore, Horsley et al. [12] observed that injury rates across professional football and elite tennis were approximately 2.0 and 2.5–2.9 injuries per player each season, respectively, which were notably higher than the estimated 0.80 injuries per player per squash season.

While Verow [18] implied that athletes should achieve adequate fitness levels before commencing squash, potentially prioritising athletic performance, playing squash in a casual and safe environment may benefit a player’s physical and mental health, regardless of skill level, and thus encourage further participation in sporting activities. Therefore, given the rising epidemics of obesity and sedentarism, squash may help to improve global cardiovascular health, MSK strength, and fitness levels. Moreover, all demographics can participate across a variety of settings, since its relatively low injury frequency, reinforced through adoption of protective equipment and effective warm-up strategies, deems it a safe, accessible, and rewarding physical activity.

Nonetheless, heat-related illness, increased body-heat storage, and thermal strain are significant health risks to consider within squash [45]. The overall rate of cardiac-related illness (or death, consequently) within squash is considered low [39]; however, players should be encouraged to statistically monitor their physical and physiological health as they advance through competitive levels of the sport to sustain a healthy and effectively functioning cardiovascular system.

Despite this, quantifiable illness data detailing rates of incidence and prevalence is limited, particularly for elite players [10], and few studies have investigated the full scope of its impact on squash athletes. However, it is important to understand the incidence and prevalence of squash-related illnesses to justify the implementation of appropriate preventative measures [10]. Therefore, future research should focus on elite squash players’ thermoregulatory responses during training and matches to address the knowledge gap regarding the extent to which heat-related illness may affect a squash player’s fitness level and recovery time and allow for the development and implementation of evidence-based prevention strategies [10].

### 4.2. Overall Injury Incidence and Prevalence

Injury incidence indicates the rate at which new injuries occur within a specified period [34], and injury prevalence quantifies the number of existing cases, both new and old, within a population at a specific point in time or over a defined period. By adopting consistent reporting metrics such as “…injuries per 365 athlete-days” or “…injuries per 1000 athlete-days,” crucial health and safety data can be compared over a large scale to enrich sport-related statistics.

Notably, there is a lack of epidemiological data concerning the incidence of squash-related concussion, despite the inherent risk of in-court collisions attributed to the “high velocity, close contact, small area, and the use of rackets” [20] (p. 231). However, one source reported that “mild concussion” was an example of a serious “no time loss” injury, which had the potential to carry severe consequences [34] (p. 213). Similarly, 15.03% of injuries affecting English professional players affected the head and spine, and all nerve injuries were associated with the head region [12]. Moreover, many other sources detailed the prevalence, severity, and mechanism of injuries affecting the head, face, eye, and dental regions, strongly implying that concussion carries a significant risk within squash. Therefore, the author calls for prompt development and implementation of comprehensive concussion guidelines for squash players worldwide, especially considering its debut in the 2028 Olympics.

### 4.3. Statistical Contributions

A meta-analysis of study findings suggested that squash-related injuries occurred at a pooled rate of 0.74 injuries per 365 athlete-days or 2.01 injuries per 1000 athlete-days. However, the high heterogeneity (I^2^ = 99.65%) denotes many methodological differences between the chosen studies, potentially influenced by the following:

- Populations: The studies investigated various demographics across all ages, such as elite professionals, elite adolescents, Indian club players, competitive Malaysian adolescents, and the general Finnish population.
- Methodologies: Some studies employed prospective cohort study designs, whereas others used retrospective surveys; others solely analysed hospital data or clinical records.
- Injury Definitions: There was great variation in what was determined an “injury” (Table 1).
- Regional Focus: While most studies focused on general MSK injuries, some were more specific to a particular body region, such as orofacial and dental injuries [20].

Additionally, many CI findings comprised wide ranges, and collation of individual study findings produced a great spectrum of incidence rates, potentially as a direct result of high heterogeneity, reflecting some quantifiable uncertainty. Therefore, while the computed results are plausible, they may not reflect an accurate incidence rate of any specific population and should thus be interpreted with academic caution. However, most studies concurred that lower limb injuries were most frequently reported.

Overall, while the meta-analysis yielded a statistically significant estimate of injury rate (*p* = 0.0285), the high heterogeneity suggests that a definitive injury rate based on current data is currently indeterminable, as it is strongly influenced by a study’s individual methodological and population choices. Therefore, future studies should employ standardised sport-based “injury” and “illness” definitions and study methodologies, akin to pre-existing International Olympic Committee (IOC) consensus statements across other sporting fields, to enhance epidemiological comparisons across the literature.

### 4.4. Comparison with Existing Literature

Calculating the rate of injury incidence allows for interpretation of an athlete’s quantifiable and time-based sports-related injury risk, as shown in Equation (1):

Injury Rate (*h*^−1^) = (Number of Injuries)/(Total Exposure Time (*h*)) × 1000. (1)

Secondly, a player’s risk of injury appeared to vary across age groups. For example, while older players [19,35], including players over 40 [36], may have suffered more frequent or severe injuries, Parkkari, Kannus, Natri, Lapinleimu, Palvanen, Heiskanen, Vuori and Jarvinen [34] contrastingly suggested that risk may decrease with age; this was supported by the physiological theory that younger players may be subjected to higher physical and physiological demands [3,32,33]. This may help to justify the findings of Horsley, O’Donnell and Leeder [12], where most injuries occurred to those aged 18–23, with a mean age of 25 years. Meyer, Van Niekerk, Prinsloo, Steenkamp and Louw [33] (p. 3) further suggested that squash is shifting “from enjoyment… to competitiveness”, which may further intensify its biomechanical demands.

Furthermore, the literature is divided on the varying abundance of acute and overuse injuries. Chard and Lachmann [35] stated that older players may sustain more acute injuries, whereas other researchers report more overuse injuries across younger players [33]. Therefore, the author advocates for the development of an appropriate risk-identifying tool, based on epidemiological data, that may be used to clinically guide a physician’s advice regarding relevant age- and skill-related risks in squash.

Methodological variations can limit comparability of study findings [3,19,34,35]; see Table 4 for a comparison of research designs per study. Reported findings may be skewed by retrospective surveys [35], which may produce recall bias. Furthermore, exclusive use of hospital findings may only report the most severe cases [19], and general surveys may comprise a much wider spectrum of injury severity.

Additionally, variations in applied terminology, such as the contrasting “time loss” definitions as adopted by Rejeb, Johnson, Vaeyens, Horobeanu, Farooq and Witvrouw [32] and Parkkari, Kannus, Natri, Lapinleimu, Palvanen, Heiskanen, Vuori and Jarvinen [34], may yield statistical inconsistencies. Consequently, future research should focus on establishing an effective standardisation strategy concerning reporting metrics to promote comparability of sporting statistics across the literature.

### 4.5. Anatomical Distribution of Squash Injuries

Squash’s high physical and technical demands can include rapid direction changes, lunging, twisting, and overhead swings. These movements can place significant stress on a player’s MSK system and produce consistent injury patterns [4,12,17,33,35]

Lower limb injuries were frequently reported, likely due to the repetitive dynamic movements and court constraints [17,35]; see Supplemental Material S8 for a full report of affected regions. Ankle and lower leg injuries were typically caused by excessive inversion, plantar flexion, or internal rotation [36]. Talabi, Olaitan, Bakinde and Onigbinde [4] suggested that ankle and lower leg injury risk can be exacerbated by a lack of lower limb fitness and donning inappropriate footwear. Knee injuries were often attributed to damage of the medial and/or lateral collateral ligaments, anterior and/or posterior cruciate ligaments, or medial and/or lateral menisci [35] and were generally more prevalent among older players [13]. Younger players tended to sustain more thigh and hamstring injuries [17], likely accredited to imbalances between bone growth and muscle flexibility [33].

Chronic shoulder injuries, usually affecting the player’s dominant arm, were shown to occur following recurrent overhead swings, often attributed to muscular overuse, rotator cuff instability, or high-speed direct trauma [33]. Similarly, elbow injuries were commonly linked with hand and wrist injuries and exacerbated by poor racket control [17,35]. Lateral epicondylitis was frequently reported following wrist overuse, highlighting the need for defined wrist musculature among squash players [17,35]. Therefore, future research could focus on wrist-specific strengthening programmes to improve performance and reduce injury risk [3].

Lower back pain was commonly reported among squash athletes, often attributed to repetitive hyperextension and rotational strain [17,33,36]. Though relatively less frequent, trauma to the eye, facial, and/or dental regions could yield highly severe consequences [17,19], often caused by ball-kicking, racket-kicking, or wall collisions [4,36]. Okhovatian and Ezatolahi [17] and Eime et al. [19] collectively highlighted the importance of donning effective and protective eyewear, irrespective of a player’s demographic or skill level.

### 4.6. Overall Certainty of Evidence

A GRADE assessment summary is provided in Supplemental Material S9, where overall certainty of evidence was commonly identified as low or very low. This was primarily due to the high frequency of retrospective studies, which generally presented a higher risk of recall bias, imprecision of injury incidence rates, and significant possibility of publication bias.

### 4.7. Strengths and Limitations of This Review

The completed PRISMA checklists accompanying this systematic review are available in Supplemental Material S10. Overall, this review benefitted from comprehensive data collection across large populations and diverse demographics. For example, studies by Horsley, O’Donnell and Leeder [12] and Eime, Zazryn and Finch [19] captured a wide spectrum of injuries through hospital records and physiotherapist-guided assessments, respectively, enhancing the accuracy and efficiency of injury assessments. Additionally, Parkkari, Kannus, Natri, Lapinleimu, Palvanen, Heiskanen, Vuori and Jarvinen [34] adopted a prospective cohort design, often considered to be more reliable than retrospective data collection, and adopted detailed and consistent key term definitions. Furthermore, insights from niche populations, such as adolescent squash players [33,34] and English professionals [12], significantly enhanced the depth and reliability of this review.

Limitations may include the use of many retrospective studies, predominantly in the form of self-reporting questionnaires; these carried the potential to produce varying levels of recall bias [3,34,35]. Similarly, studies relying on interviews could have provided less diagnostically accurate data than those utilising standardised hospital-based criteria due to variations in diagnostic protocols [34]. Furthermore, where studies (n = 4) did not indicate the total number of exposure days across the population, it was impossible to calculate the rate of injury incidence over time [12,32,35]. Additionally, studies carried out in diverse countries or relying on self-referral statistics may not have fully identified all injuries to a professional standard, producing a poor estimate of the global injury burden [35].

### 4.8. Recommendations for Future Research

Despite the inherent risk of significant orofacial injuries within squash, many players reported that they chose not to wear appropriate protective eyewear or mouthguards [20]. However, studies by Persic [20] and Eime [19] strongly recommended utilisation of these protective measures across all squash demographics. Furthermore, the author calls for a greater emphasis on squash-specific fitness and conditioning programmes to reduce the prevalence of overuse injuries [33]. Additionally, squash players should be educated about the health risks associated with playing while injured and encouraged to take appropriate recovery time to reduce the risk of chronic injury [13] and seek higher levels of medical attention when necessary. Additionally, first-aid education should be made increasingly accessible to all squash players and coaches [35].

Furthermore, future research should adopt consistent exposure-based metrics definitions regarding sport-related injuries and illnesses [34,37]. Longitudinal research could provide epidemiological insights into the long-term impact of age, training load, and competitive level variations on injury and illness risk [12,33,34,35].

Additionally, stricter rule enforcement may reduce the prevalence of severe injuries and illnesses [4,20]. Combining healthy fitness levels with appropriate warm-up strategies may help in alleviating the harmful physiological effects of heat strain and cardiovascular stress, particularly among older populations [4,37]. Building upon the findings of Northcote, Evans and Ballantyne [39], Finch and Eime [37] suggested that all squash players should undergo a thorough cardiovascular risk pre-assessment to assess individual suitability and degree of personal risk and receive education regarding red-flag symptoms; similarly, Parkkari, Kannus, Natri, Lapinleimu, Palvanen, Heiskanen, Vuori and Jarvinen [34] recommended that those with a personal or family history of cardiovascular pathology should receive a clinical assessment before playing.

However, there was a lack of comprehensive data on squash-related illnesses beyond cardiovascular stress and heat strain, and many relevant studies were insufficient or outdated [37]. Furthermore, many studies excluded illness data, and some only reported severe anomalous outcomes such as squash-related sudden deaths. There are currently no published studies investigating the broad topic of illness incidence and prevalence within squash [37], highlighting the need for deeper epidemiological research in this area to further support the development of global fitness and well-being via the implementation of appropriate prevention protocols [33]. For instance, such measures could prioritise the execution of adequate warm-up regimes to address the high prevalence of MSK-related injuries; similarly, implementing compulsory recovery periods between matches during squash tournaments could positively contribute to the alleviation of fatigue-induced decline in biomechanical performance.

Consequently, future studies should focus on the distinct injury and illness patterns across age, gender, and ability levels to enable reassessment of current diagnostic approaches, sporting recommendations, and medical treatments following sporting injury [37]. Coaches and clubs should maintain accurate injury- and illness-related tracking data and adopt evidence-based support systems to improve the quality of injury recovery and support the global implementation of effective prevention strategies [3].

## 5. Conclusions

This systematic review and meta-analysis highlighted the increasing significance of squash-related injuries and illnesses across the globe. The literature commonly identified squash as intense, dynamic, and challenging, often attributed to the agility required within its fast-paced environment. There was a wide spectrum of injury data across various ages, genders, and skill levels, notably of MSK trauma affecting the lower limbs, upper limbs, back, and face. Chronic overuse injuries among younger athletes, alongside a low uptake of orofacial protective measures, highlighted the ongoing necessity for enhanced sporting education, strength and conditioning regimes, and injury prevention strategies within squash. While there is a globally growing concern of heat strain and cardiovascular stress, particularly in advance of squash’s introduction to the Olympic Games in 2028, illness-related data remains limited. Hence, the author calls for future studies to progressively address these topics using standardised terminologies, methodologies, and reporting metrics to encourage a safer, more inclusive, and performance-focused future within the world of squash.

## Figures and Tables

**Table 1:** Comparative summary of squash-related injury definitions, classifications, measurement units, and reported rates across studies.

Source	Definition of Injury	Injury Classifications	Injury Measurement Unit	Calculated Squash Injury Rate
Jhamb and Singh (2022) [13]	No specific definition provided: injuries reported individually by participants via structured questionnaire. Injury was considered significant if the player had to miss at least two weeks of squash as a result.	If a player had sustained multiple injuries, information was only sought on the most severe injury.	Percentage of population who had sustained a squash injury requiring medical attention and two weeks off. Average time to recovery (months).	86% of population injured (N = 104/120). Average time to recovery = 5.8 months. No exposure time to calculate rate.
Horsley et al. (2020) [12]	“…any musculoskeletal condition that prevented the player from participating in either training or competition for more than 24 h.” (p. 2)	Injuries were coded using a modified OSICS-10.	Injuries per athlete over the study’s duration (190 months). Percentage by body region.	8.83 injuries per player over 11 years (N = 592/67). No exposure time to calculate rate.
Rejeb et al. (2017) [32]	“…a physical complaint requiring the attention of the medical staff resulting from either sports training, strengthening and conditioning training, or a competition.” (p. 497)	No time-loss injury: Medical attention required; no missed full training session or competition. Time-loss injury: Unable to fully take part in a training session or competition. Traumatic injury: Injury resulting from a specific and identifiable mechanism; acute onset. Overuse injury: Injury resulting from insidious onset without a clear mechanism. Growth condition injury: Unique to young athletes, due to increased involvement in sports activities.	Injuries per 1000 h of exposure.	8.5 injuries per 1000 h. Highest rate of all activities investigated.
Sankaravel et al. (2017) [3]	“…any pain and/or disability sustained by the squash player during competitions or training sessions resulting in time lost from sports participation in the last 12 months.” (p. 1134)	Injuries were classified based on the nine anatomical sites of the SNQ: neck, shoulder, elbow, wrist/hand, upper back, lower back, hip/thigh, knee, and ankle/feet.	Percentage of players with MSK symptoms over the last 12 months.	83.3% had symptoms over the last 12 months (N = 50/60). No exposure time to calculate rate.
Talabi et al. (2012) [4]	No specific definition provided: injuries were reported individually by participants via structured questionnaire.	Soft tissue (85.70%); sprain (10.30%); fracture (2.70%); others (1.30%).	Percentage of injury type, percentage by body region.	No exposure time to calculate rate.
Okhovatian and Ezatolahi (2009) [17]	No specific definition provided; injuries were reported individually by participants via structured questionnaire.	Detailed report of squash injuries.	Percentage of players sustaining at least one injury over the previous two years.	79% injured within the last two years (N = 41/52); no exposure time to calculate rate.
Meyer et al. (2007) [33]	“…one that occurred during practice or competition resulting from a traumatic incident.” (p. 5)	“…encompassed overuse injuries not initiated by a specific traumatic incident, but causing symptoms including pain or swelling while or after playing squash.” (p. 5)	Percentage of players injured over the previous four weeks; injuries per 1000 h of participation.	29% of players were injured in the last four weeks (N = 31/106), where 48 injuries were reported overall; 0.45 injuries per 1000 playing hours.
Persic et al. (2006) [20]	No specific definition was provided; orofacial injuries were reported individually by participants via structured questionnaire.	All reported injuries, self-experienced (N = 27) and observed (N = 142): • Crown fracture (N = 109); • Avulsion (N = 48); Dislocation (N = 12).	Percentage of players who have ever sustained an orofacial or a dental injury.	4.5% suffered dental trauma (N = 27/600); 20.4% observed dental trauma (N = 133/653); no exposure time to calculate rate.
Parkkari et al. (2004) [34]	“…a new acute trauma or overuse injury that caused a significant complaint to the subject.” (p. 210)	Level I: No activity time lost Level II: Time lost (at least one sport/leisure activity session missed) Level III: Time lost (at least one day of work/activity missed)	Injuries per 1000 person-years; Injuries per 1000 active persons at risk, Injuries per 1000 h of exposure.	18.3 injuries per 1000 playing hours (95% CI 11.4–29.4); highest rate of all activities investigated.
Eime et al. (2003) [19]	Only covered severe injuries, which were defined “…as those warranting medical treatment at a hospital setting.” (p. 245)	VAED (hospital admissions); VEMD (ED presentations).	Injured players per 100,000 players.	ED = 58.5 injured players per 100,000 players.
Chard and Lachmann (1987) [35]	No specific definition provided; the clinic assessed both acute (within 48 h) and chronic injuries but generally excluded lacerations, fractures, and eye and head injuries which were treated acutely by the Accident Service.	Acute traumatic injuries (80%); overuse injuries (20%).	Percentage of total injuries.	59.0% sustained squash injuries (N = 372/631); no exposure time to calculate rate.
Berson et al. (1981) [36]	No specific definition provided; injuries were reported individually by participants via structured questionnaire.	Disabling injury: “Any injury which kept the player out of action for more than two weeks.” (p. 104)	Percentage of injured players; percentage of disabling injuries.	Injury rate = 44.5% (N = 69/155); no exposure time to calculate rate.

Abbreviations: CI, Confidence Interval; ED, Emergency Department; MSK, Musculoskeletal; N, Number of participants/injuries; OSICS, Orchard Sports Injury Classification System; SNQ, Standardised Nordic Questionnaire; VAED, Victorian Admitted Episodes Dataset; VEMD, Victorian Emergency Minimum Dataset.

**Table 2:** Summary of squash-specific data reporting the total number of days spent playing squash, active participants, and squash-related injuries.

Source	Total Number of Days	Total Number of Players	Total Number of Injuries
Horsley et al. (2020) [12]	4015	67	592
Rejeb et al. (2017) [32]	1610	18	~153
Sankaravel et al. (2017) [3]	365	60	50
Okhovatian and Ezatolahi (2009) [17]	730	52	91
Meyer et al. (2007) [33]	28	106	48
Parkkari et al. (2004) [34]	365	27	17
Eime et al. (2003) [19]	2190	389	389
Chard and Lachmann (1987) [35]	2920	372	372

**Table 3:** Statistical indicators of heterogeneity across studies.

Symbol	Description
k	Number of studies included in the meta-analysis.
$Q\left(df\right)$	Cochran’s Q-statistic to test heterogeneity (degrees of freedom in parentheses).
$I^{2}$	Percentage of variability across studies due to heterogeneity rather than random error.
$\tau^{2}$	Estimate of between-study variance (tau-squared).

**Table 4:** Methodological approaches used in each study.

Source	Study Design
Jhamb and Singh (2022) [13]	Cross-sectional observational study
Horsley et al. (2020) [12]	Cross-sectional survey
Rejeb et al. (2017) [32]	Prospective cohort study
Sankaravel et al. (2017) [3]	Cross-sectional study
Talabi et al. (2014) [4]	Descriptive cross-sectional study
Okhovatian and Ezatolahi (2009) [17]	Cross-sectional study
Meyer et al. (2007) [33]	Cross-sectional survey
Persic et al. (2006) [20]	Cross-sectional observational study
Parkkari et al. (2004) [34]	Large-scale population-based retrospective study
Eime et al. (2003) [19]	Retrospective epidemiological study
Chard and Lachmann (1987) [35]	Retrospective analysis
Berson et al. (1981) [36]	Retrospective survey

## Data Availability

The original contributions presented in this study are included in the article/Supplementary Material. Further inquiries can be directed to the corresponding author.

## Supplementary Materials

The following supporting information can be downloaded at https://www.mdpi.com/article/10.3390/sports14020079/s1, Supplemental Material S1: PROSPERO Registration Document; Supplemental Material S2: Database Search Strategy; Supplemental Material S3: Self-Created Data Extraction Template; Supplemental Material S4: National Institute of Health Quality Assessment Tool and Results; Supplemental Material S5: Study Characteristics; Supplemental Material S6: Key Findings and Risk Factors; Supplemental Material S7: Reported Distribution of Squash Injuries; Supplemental Material S8: Categorisation of Reported Lower Limb Injuries; Supplemental Material S9: Certainty of Evidence (GRADE) Assessment. Supplemental Material S10. Completed PRISMA Checklists for Reporting of Systematic Reviews.

## Abbreviations

The following abbreviations are used in this manuscript:

CI	Confidence Interval
ED	Emergency Department
GRADE	Grading of Recommendations Assessment, Development and Evaluation
IOC	International Olympic Committee
MSK	Musculoskeletal
NHBLI	National Heart, Lung, and Blood Institute
OSICS	Orchard Sports Injury Classification System
PRISMA	Preferred Reporting Items for Systematic Reviews and Meta-Analyses
PROSPERO	International prospective register of systematic reviews
RR	Relative Risk
SNQ	Standardised Nordic Questionnaire
SPIDER	Sample, Phenomenon of Interest, Design, Evaluation, Research type
VAED	Victorian Admitted Episodes Dataset
VEMD	Victorian Emergency Minimum Dataset
