# Supplementary material for: Squash Under Strain: A Systematic Review and Meta-Analysis of Injuries and Illnesses in Squash Players
Source: Sports (Basel). 2026 Feb 11;14(2):79. doi: 10.3390/sports14020079 (PMC12944960; doi:10.3390/sports14020079)
Supplement: Supplementary file 1 [file sports-14-00079-s001.zip › sports-4040985-supplementary.pdf]

sports

MDPI

Squash Under Strain: A Systematic Review and Meta-Analysis of Injuries and Illnesses in Squash Players

Supplemental Materials

Supplemental Material S1. PROSPERO Registration Document. **2**

Supplemental Material S2. Database Search Strategies. **8**

Supplemental Material S2(a). MEDLINE Search Strategy. 8

Supplemental Material S2(b). Embase Search Strategy. 8

Supplemental Material S2(c). Web of Science Core Collection Search Strategy 8

Supplemental Material S3. Self-Created Data Extraction Template. **9**

Supplemental Material S4. National Institute of Health Quality Assessment Tool for Observational Cohort and Cross-Sectional Studies (National Institute of Health, 2021). **10**

Supplemental Material S4(a). Questions contained within the National Institute of Health Quality Assessment Tool for Observational Cohort and Cross-Sectional Studies (National Institute of Health, 2021). 10

Supplemental Material S4(b). Manual quality assessment of included studies using the National Institute of Health Quality Assessment Tool for Observational Cohort and Cross-Sectional Studies (National Institute of Health, 2021). 14

Supplemental Material S5. Study Characteristics. **15**

Supplemental Material S6. Key Findings and Risk Factors. **16**

Supplemental Material S7. Reported Distribution of Squash Injuries. **18**

Supplemental Material S7(a). Distribution of Squash Injuries by Anatomical Region. 18

Supplemental Material S7(b). Distribution of Squash Injuries by Tissue Type. 19

Supplemental Material S8. Categorisation of Reported Lower Limb Injuries by Anatomical Region. **20**

Supplemental Material S9. Certainty of Evidence Assessment. **21**

Supplemental Material S9(a). GRADE Assessment Table 21

Supplemental Material S9(b). Downgrading Justification.. 21

Supplemental Material S10. Completed PRISMA Checklists for Reporting of Systematic Reviews. **23**

Supplemental Material S10(a). PRISMA 2020 Checklist. 23

Supplemental Material S10(b). PRISMA 2020 for Abstracts Checklist. 27

Supplemental Material S1. PROSPERO Registration Document.

NIHR | National Institute for Health and Care Research

PROSPERO
International prospective register of systematic reviews

A Systematic Review of Squash Injuries and Illnesses

Rachel McCartney, Neil Heron

Citation
Rachel McCartney, Neil Heron. A Systematic Review of Squash Injuries and Illnesses. PROSPERO 2025 CRD420251081709. Available from https://www.crd.york.ac.uk/PROSPERO/view/CRD420251081709.

REVIEW TITLE AND BASIC DETAILS

Review title
A Systematic Review of Squash Injuries and Illnesses

Condition or domain being studied
Sports; Sports Injury; Injury; Illness; Musculoskeletal injury; Hyperthermia; Exercise; Cognitive function

This systematic review and meta-analysis will focus on injuries and illnesses associated with squash and their consequent impact on a player's performance and well-being.

Rationale for the review
Squash is a dynamic sport with a growing global popularity, yet it presents a significant injury risk. Despite this, there are few papers providing comprehensive, systematically analysed data on the full spectrum of squash-related health issues. Many existing studies vary in methodology, definitions, and reporting standards, which makes it difficult to compare findings. In addition to this, many studies are outdated and do not reflect changes in squash gameplay that may influence injury risk.

This review aims to address these gaps by synthesising current evidence via systematic review and meta-analysis using standardised metrics (such as 'injuries per 1,000 hours') and evaluation of the sport's physical and physiological demands. By doing so, it may help to contribute towards the development of evidence-based injury prevention protocols, equipment design, and new safety policies.

Review objectives
The primary aim of this study is to perform a systematic review and meta-analysis of the global patterns of squash-related injuries and illnesses across all demographics, addressing the following specific questions:

1. What are the most common squash-related injuries and illnesses?
2. How do these conditions affect athletic performance, cognitive function, and overall well-being?
3. What gaps exist in the current literature, and how can future research address them?

Therefore, this review aims to provide a comprehensive and up-to-date synthesis of the literature, using standardised reporting metrics (such as 'injuries per 1,000 hours') to inform future research in squash and wider sporting contexts.

Keywords
Squash; Sports injury; Acute illness; Chronic illness; Musculoskeletal pain; Heat strain; Heat-related illness; Injury prevention; Epidemiology; Athletic performance; Sports nutrition; Physiological stress; Protective equipment; Systematic review; Injury surveillance; Sports medicine; Well-being; Racquet sports; Cognitive function; Injury risk factors; Evidence-based prevention

Country
Northern Ireland

ELIGIBILITY CRITERIA

Population

Included

- Squash players of any age, gender, or competitive level.
- Epidemiologic studies reporting on squash-related injuries, illnesses, or health conditions.
- Studies that provide detailed data on squash-related injuries, illnesses or health conditions.
- Studies that explore the impact of these conditions on a squash player's performance, well-being, and/or career longevity.

Excluded

- Studies that do not distinguish squash players from participants of other sports.
- Studies that do not report health outcomes.
- Studies without human participants.
- Studies where data specific to squash players cannot be extracted separately.

Intervention(s) or exposure(s)

Included
High Intensity Exercise; Low Intensity Exercise; Training; Continuous physiological monitoring; Preventing Injury; Environmental intervention; Personal Protective Equipment; Nutrition Care; Hyperthermia Treatment

This review focuses on exposures rather than interventions, specifically evaluating the physical, environmental, and physiological factors contributing to squash-related injuries and illnesses:

- Biomechanical and physiological demands of squash.
- Environmental conditions and equipment-related factors.
- Training and match workload.
- Rule changes that may influence player workload and injury risk.
- Travel-related stressors and/or nutritional deficiencies.

Excluded

- Studies that do not isolate squash-specific data.
- Studies focusing solely on treatment or rehabilitation.

Comparator(s) or control(s)
This review does not have any comparators

Study design
Both randomized and nonrandomized study types will be included.

Included
Inclusion Criteria:

- Observational studies, including prospective and retrospective cohort studies.
- Mixed-methods studies combining quantitative and qualitative data regarding sport-related injuries and illnesses.

Excluded
Exclusion Criteria:

- Editorials, opinion pieces, and reviews without original data.
- Case reports and case series that discuss anomalous squash-related injuries or illnesses.
- Qualitative studies that explore perspectives instead of confirmed quantitative data.
- Studies where squash data cannot be extracted separately.
- Studies without human participants.

Context
This review will include studies including:

- Competitive and recreational squash environments.
- Indoor squash courts.
- Clinical settings where squash-related injuries or illnesses are reported.
- All demographics across the global population.

Exclusion Criteria:

- Simulated studies conducted without real-world squash exposure.
- Studies where the setting is not relevant to squash.

TIMELINE OF THE REVIEW

Date of first submission to PROSPERO
10 July 2025

Review timeline
Start date: 2 June 2025. End date: 15 September 2025.

Date of registration in PROSPERO
15 August 2025

AVAILABILITY OF FULL PROTOCOL

Availability of full protocol
A full protocol has been written and uploaded to PROSPERO. The protocol will be made available after the review is completed.

SEARCHING AND SCREENING

Search for unpublished studies
Only published studies will be sought.

Main bibliographic databases that will be searched
The main databases to be searched are *Embase - Embase via Ovid*, *MEDLINE*.

Other important or specialist databases that will be searched
Web of Science Core Collection

Search language restrictions
The review will only include studies published in English.

Search date restrictions
There are no search date restrictions.

Other methods of identifying studies
Other studies will be identified by: *looking through all the articles that cite the papers included in the review ("snowballing")*, *reference list checking* and *searching dissertation and thesis databases*.

Link to search strategy
A full search strategy has been uploaded to PROSPERO. The PDF may be accessed through this link https://www.crd.york.ac.uk/PROSPEROFILES/45a3cd2aa87c5a7f1c3bca2d6ae849ea.pdf.

Selection process
Studies will be screened independently by at least two people (or person/machine combination) with a process to resolve differences.

Other relevant information about searching and screening
Papers will be screened using Rayyan AI, an online platform used to support collaboration and communication between reviewers.

DATA COLLECTION PROCESS

Data extraction from published articles and reports
Data will be extracted independently by at least two people (or person/machine combination) with a process to resolve differences.

Authors will not be contacted for further information.

Study risk of bias or quality assessment
Risk of bias will be assessed using:

NHLBI Study Quality Assessment Tools.

Data will be assessed by one person (or a machine) and checked by at least one other person (or machine).

Additional information will **not** be sought from study investigators if required information is unclear or unavailable in the study publications/reports.

Reporting bias assessment

Potential methods may include, but are not limited to:

- Funnel plot analysis.
- Statistical tests for funnel plot asymmetry.
- Selective reporting within studies.
- Outcome Reporting Bias Tool (ORBIT).
- Sensitivity analyses.
- GRADE consideration.

Certainty assessment

The GRADE (Grading of Recommendations Assessment, Development and Evaluation) approach will be used, based on the following:

- Risk of Bias
- Inconsistency
- Indirectness
- Imprecision
- Publication Bias

Each outcome will be rated as one of the following:

- High certainty
- Moderate certainty
- Low certainty
- Very low certainty

OUTCOMES TO BE ANALYSED

Main outcomes

Primary Outcomes

Injury and Illness Incidence

- Generally defined as the number of squash-related injuries or illnesses reported per study population.
- Reported as frequency counts or incidence rates (such as 'injuries per 1,000 hours of exposure').
- Comparison may be made using incidence rate ratio (IRR) or between raw frequency data.

Injury and Illness Type and Location

- Considered as the classification of injuries and/or illnesses by anatomical region and type, based upon a clinical diagnosis or predefined classification.
- Important considerations include their proportional distribution and/or frequency comparisons.

Severity and Recovery Time

- Considered as the severity of injury and/or illness and time lost from training or competition.
- Relevant measurements may include the number of days of sport missed, days of hospitalisation, or the time taken to return to play from the onset of injury and/or illness; calculations may include average recovery times or severity grades.

Secondary Outcomes

Risk Factors and Mechanisms

- Considered to be a spectrum of factors which contribute to a player's squash-related injury or illness.
- Measurements may include reported associations or risk models, either preceding or during each event.

Effectiveness of Prevention Strategies

- Considered as the implementation and outcomes of injury and/or illness prevention interventions.
- Measurements may include calculating reduction in injury/illness rates: either pre- or post-intervention or over a follow-up period.

Additional outcomes

Additional outcomes may comprise, but are not limited to:

- Thermoregulatory strain
- Cognitive function
- Player knowledge and awareness (including use of protective equipment)
- Training and match load
- Career longevity and retirement factors

PLANNED DATA SYNTHESIS

Strategy for data synthesis

Where studies report homogeneous and detailed statistical data, meta-analysis will be conducted using a random-effects model. For dichotomous outcomes, effect sizes may be expressed as risk or odds ratios with 95% confidence intervals, and standardised mean differences can be used for continuous outcomes. If meta-analysis is not possible, alternative suitable statistical methods will be considered on a case-by-case basis.

CURRENT REVIEW STAGE

Stage of the review at this submission

Review stage	Started	Completed
Pilot work	✓	
Formal searching/study identification	✓	
Screening search results against inclusion criteria	✓	
Data extraction or receipt of IPD		
Risk of bias/quality assessment		
Data synthesis		

Review status

The review is currently planned or ongoing.

Publication of review results

Results of the review will be published in English.

REVIEW AFFILIATION, FUNDING AND PEER REVIEW

Review team members

Miss Rachel McCartney (review guarantor and contact) Queen's University Belfast. Northern Ireland.

No conflict of interest declared.

Dr Neil Heron. Queen's University Belfast. Northern Ireland.

No conflict of interest declared.

Named contact

Miss Rachel McCartney (rmccartney09@qub.ac.uk). Queen's University Belfast. Northern Ireland.

Review affiliation

Queen's University Belfast

Funding source

Review has no funding and no agreed support from an academic institution and is done in authors' own time.

Peer review

There has been no peer review of this planned review.

ADDITIONAL INFORMATION

Additional information

This review has been developed as part of the MSc in Clinical Anatomy at Queen's University Belfast.

Review conflict of interest

Declared individual interests are recorded under team member details.. No additional interests are recorded for this review.

Medical Subject Headings

Racquet Sports; Athletes; Athletic Injuries; Facial Injuries; Tooth Injuries; Craniocerebral Trauma; Travel-Related Illness; Hyperthermia, Induced; Hot Temperature; Malnutrition; Sports Medicine; Travel; Protective Devices; Athletic Performance; Cognition; Policy; Humans; Demography

SIMILAR REVIEWS

Check for similar records already in PROSPERO

PROSPERO identified a number of existing PROSPERO records that were similar to this one (last check made on 27 June 2025). These are shown below along with the reasons given by that the review team for the reviews being different and/or proceeding.

- Squash-related injuries: A systematic literature review [published 15 February 2024] [CRD42024506169]. The review was acknowledged as **similar** but the authors opted to continue because *the review looks at additional or different outcomes, the review will be more up to date, the review uses improved methods*
- Effect of Homeopathic Interventions in Sports Medicine: A Systematic Review and Meta-analysis [published 3 July 2023] [CRD42023434119]. The review was judged **not to be similar**
- Effects of Integrated Neuromuscular Training on Improving Athletes' Jumping Performance and Injury Prevention: A Meta-analysis [published 15 January 2024] [CRD42024496230]. The review was judged **not to be similar**

PROSPERO version history

- Version 1.0, published 15 Aug 2025

Disclaimer

The content of this record displays the information provided by the review team. PROSPERO does not peer review registration records or endorse their content.

PROSPERO accepts and posts the information provided in good faith; responsibility for record content rests with the review team. The guarantor for this record has affirmed that the information provided is truthful and that they understand that deliberate provision of inaccurate information may be construed as scientific misconduct.

PROSPERO does not accept any liability for the content provided in this record or for its use. Readers use the information provided in this record at their own risk.

Any enquiries about the record should be referred to the named review contact

Supplemental Material S2. Database Search Strategies.

Supplemental Material S2(a). MEDLINE Search Strategy.

1	squash.mp.
2	Wounds, Nonpenetrating/ or injur*.mp. or "Wounds and Injuries"/
3	Travel-Related Illness/ or Critical Illness/ or illness*.mp.
4	nutrition*.mp.
5	Eye Injuries, Penetrating/ or Eye Injuries/ or eye trauma.mp.
6	Head Injuries, Closed/ or Brain Concussion/ or Brain Injuries/ or Craniocerebral Trauma/ or head trauma*.mp. or Brain Injuries, Traumatic/
7	Abdominal Injuries/ or abdomen trauma.mp.
8	2 or 3 or 4 or 5 or 6 or 7
9	1 and 8
10	limit 9 to (abstracts and english language and humans)

Supplemental Material 2(b). Embase Search Strategy.

1	squash player/ or squash.mp. or "squash (sport)"/
2	injury/ or traumatic brain injury/ or injur*.mp.
3	illness*.mp. or critical illness/
4	sport nutrition/ or nutrition*.mp.
5	eye trauma.mp. or eye injury/
6	head trauma.mp. or head injury/
7	abdomen trauma.mp. or abdominal injury/
8	limb trauma.mp. or limb injury/
9	2 or 3 or 4 or 5 or 6 or 7 or 8
10	1 and 9
11	limit 10 to (abstracts and human and english language)
12	squash/
13	11 not 12

Supplemental Material S2(c). Web of Science Core Collection Search Strategy.

squash (All Fields) and **illness* or injur* or trauma* or musculoskeletal or nutrition* or travel* or "time zone*"** (All Fields) and **sport* or athlet* or professional** (All Fields)

Supplemental Material S3. Self-Created Data Extraction Template.

Details of Data Required		Extracted Study Data
Study Identification:	Source	-
Study Characteristics:	Methodology	-
	Setting and Location	-
	Number of Participants	-
	Age Range of Participants	-
Definitions and Measurement:	Definition of Injury	-
	Injury Measurement Unit	-
	Definition of Illness	-
Key Findings:	Injury Classifications	-
	Details of Injury or Illness	-
	Risk Factors	-
	Calculated Squash Injury Rate	-
Quality Assessment:	NIH Quality Score (n=14)	-

Abbreviations: NIH, National Institutes of Health.

Supplemental Material S4. National Institute of Health Quality Assessment Tool for Observational Cohort and Cross-Sectional Studies (National Institute of Health, 2021).

Supplemental Material S4(a). Questions contained within the National Institute of Health Quality Assessment Tool for Observational Cohort and Cross-Sectional Studies (National Institute of Health, 2021).

National Heart, Lung, and Blood Institute

Quality Assessment Tool for Observational Cohort and Cross-Sectional Studies

Criteria	Yes	No	Other (CD, NR, NA)*
1. Was the research question or objective in this paper clearly stated?			
2. Was the study population clearly specified and defined?			
3. Was the participation rate of eligible persons at least 50%?			
4. Were all the subjects selected or recruited from the same or similar populations (including the same time period)? Were inclusion and exclusion criteria for being in the study prespecified and applied uniformly to all participants?			
5. Was a sample size justification, power description, or variance and effect estimates provided?			
6. For the analyses in this paper, were the exposure(s) of interest measured prior to the outcome(s) being measured?			
7. Was the timeframe sufficient so that one could reasonably expect to see an association between exposure and outcome if it existed?			
8. For exposures that can vary in amount or level, did the study examine different levels of the exposure as related to the outcome (e.g., categories of exposure, or exposure measured as continuous variable)?			
9. Were the exposure measures (independent variables) clearly defined, valid, reliable, and implemented consistently across all study participants?			
10. Was the exposure(s) assessed more than once over time?			
11. Were the outcome measures (dependent variables) clearly defined, valid, reliable, and implemented consistently across all study participants?			
12. Were the outcome assessors blinded to the exposure status of participants?			
13. Was loss to follow-up after baseline 20% or less?			
14. Were key potential confounding variables measured and adjusted statistically for their impact on the relationship between exposure(s) and outcome(s)?			

Quality Rating (Good, Fair, or Poor) (see guidance)
Rater #1 initials:
Rater #2 initials:
Additional Comments (If POOR, please state why):

*CD, cannot determine; NA, not applicable; NR, not reported

Guidance for Assessing the Quality of Observational Cohort and Cross-Sectional Studies

The guidance document below is organized by question number from the tool for quality assessment of observational cohort and cross-sectional studies.

Question 1. Research question

Did the authors describe their goal in conducting this research? Is it easy to understand what they were looking to find? This issue is important for any scientific paper of any type. Higher quality scientific research explicitly defines a research question.

Questions 2 and 3. Study population

Did the authors describe the group of people from which the study participants were selected or recruited, using demographics, location, and time period? If you were to conduct this study again, would you know who to recruit, from where, and from what time period? Is the cohort population free of the outcomes of interest at the time they were recruited?

An example would be men over 40 years old with type 2 diabetes who began seeking medical care at Phoenix Good Samaritan Hospital between January 1, 1990 and December 31, 1994. In this example, the population is clearly described as: (1) who (men over 40 years old with type 2 diabetes); (2) where (Phoenix Good Samaritan Hospital); and (3) when (between January 1, 1990 and December 31, 1994). Another example is women ages 34 to 59 years of age in 1980 who were in the nursing profession and had no known coronary disease, stroke, cancer, hypercholesterolemia, or diabetes, and were recruited from the 11 most populous States, with contact information obtained from State nursing boards.

In cohort studies, it is crucial that the population at baseline is free of the outcome of interest. For example, the nurses' population above would be an appropriate group in which to study incident coronary disease. This information is usually found either in descriptions of population recruitment, definitions of variables, or inclusion/exclusion criteria.

You may need to look at prior papers on methods in order to make the assessment for this question. Those papers are usually in the reference list.

If fewer than 50% of eligible persons participated in the study, then there is concern that the study population does not adequately represent the target population. This increases the risk of bias.

Question 4. Groups recruited from the same population and uniform eligibility criteria

Were the inclusion and exclusion criteria developed prior to recruitment or selection of the study population? Were the same underlying criteria used for all of the subjects involved? This issue is related to the description of the study population, above, and you may find the information for both of these questions in the same section of the paper.

Most cohort studies begin with the selection of the cohort; participants in this cohort are then measured or evaluated to determine their exposure status. However, some cohort studies may recruit or select exposed participants in a different time or place than unexposed participants, especially retrospective cohort studies–which is when data are obtained from the past (retrospectively), but the analysis examines exposures prior to outcomes. For example, one research question could be whether diabetic men with clinical depression are at higher risk for cardiovascular disease than those without clinical depression. So, diabetic men with depression might be selected from a mental health clinic, while diabetic men without depression might be selected from an internal medicine or endocrinology clinic. This study recruits groups from different clinic populations, so this example would get a "no."

However, the women nurses described in the question above were selected based on the same inclusion/exclusion criteria, so that example would get a "yes."

Question 5. Sample size justification

Did the authors present their reasons for selecting or recruiting the number of people included or analyzed? Do they note or discuss the statistical power of the study? This question is about whether or not the study had enough participants to detect an association if one truly existed.

A paragraph in the methods section of the article may explain the sample size needed to detect a hypothesized difference in outcomes. You may also find a discussion of power in the discussion section (such as the study had 85 percent power to detect a 20 percent increase in the rate of an outcome of interest, with a 2-sided alpha of 0.05). Sometimes estimates of variance and/or estimates of effect size are given, instead of sample size calculations. In any of these cases, the answer would be "yes."

However, observational cohort studies often do not report anything about power or sample sizes because the analyses are exploratory in nature. In this case, the answer would be "no." This is not a "fatal flaw." It just may indicate that attention was not paid to whether the study was sufficiently sized to answer a prespecified question–i.e., it may have been an exploratory, hypothesis-generating study.

Question 6. Exposure assessed prior to outcome measurement

This question is important because, in order to determine whether an exposure causes an outcome, the exposure must come before the outcome.

For some prospective cohort studies, the investigator enrolls the cohort and then determines the exposure status of various members of the cohort (large epidemiological studies like Framingham used this approach). However, for other cohort studies, the cohort is selected based on its exposure status, as in the example above of depressed diabetic men (the exposure being depression). Other examples include a cohort identified by its exposure to fluoridated drinking water and then compared to a cohort living in an area without fluoridated water, or a cohort of military personnel exposed to combat in the Gulf War compared to a cohort of military personnel not deployed in a combat zone.

With either of these types of cohort studies, the cohort is followed forward in time (i.e., prospectively) to assess the outcomes that occurred in the exposed members compared to nonexposed members of the cohort. Therefore, you begin the study in the present by looking at groups that were exposed (or not) to some biological or behavioral factor, intervention, etc., and then you follow them forward in time to examine outcomes. If a cohort study is conducted properly, the answer to this question should be "yes," since the exposure status of members of the cohort was determined at the beginning of the study before the outcomes occurred.

For retrospective cohort studies, the same principal applies. The difference is that, rather than identifying a cohort in the present and following them forward in time, the investigators go back in time (i.e., retrospectively) and select a cohort based on their exposure status in the past and then follow them forward to assess the outcomes that occurred in the exposed and nonexposed cohort members. Because in retrospective cohort studies the exposure and outcomes may have already occurred (it depends on how long they follow the cohort), it is important to make sure that the exposure preceded the outcome.

Sometimes cross-sectional studies are conducted (or cross-sectional analyses of cohort-study data), where the exposures and outcomes are measured during the same timeframe. As a result, cross-sectional analyses provide weaker evidence than regular cohort studies regarding a potential causal relationship between exposures and outcomes. For cross-sectional analyses, the answer to Question 6 should be "no."

Question 7. Sufficient timeframe to see an effect

Did the study allow enough time for a sufficient number of outcomes to occur or be observed, or enough time for an exposure to have a biological effect on an outcome? In the examples given above, if clinical depression has a biological effect on increasing risk for CVD, such an effect may take years. In the other example, if higher dietary sodium increases BP, a short timeframe may be sufficient to assess its association with BP, but a longer timeframe would be needed to examine its association with heart attacks.

The issue of timeframe is important to enable meaningful analysis of the relationships between exposures and outcomes to be conducted. This often requires at least several years, especially when looking at health outcomes, but it depends on the research question and outcomes being examined.

Cross-sectional analyses allow no time to see an effect, since the exposures and outcomes are assessed at the same time, so those would get a "no" response.

Question 8. Different levels of the exposure of interest

If the exposure can be defined as a range (examples: drug dosage, amount of physical activity, amount of sodium consumed), were multiple categories of that exposure assessed? (for example, for drugs: not on the medication, on a low dose, medium dose, high dose; for dietary sodium, higher than average U.S. consumption, lower than recommended consumption, between the two). Sometimes discrete categories of exposure are not used, but instead exposures are measured as continuous variables (for example, mg/day of dietary sodium or BP values).

In any case, studying different levels of exposure (where possible) enables investigators to assess trends or dose-response relationships between exposures and outcomes–e.g., the higher the exposure, the greater the rate of the health outcome. The presence of trends or dose-response relationships lends credibility to the hypothesis of causality between exposure and outcome.

For some exposures, however, this question may not be applicable (e.g., the exposure may be a dichotomous variable like living in a rural setting versus an urban setting, or vaccinated/not vaccinated with a one-time vaccine). If there are only two possible exposures (yes/no), then this question should be given an "NA," and it should not count negatively towards the quality rating.

Question 9. Exposure measures and assessment

Were the exposure measures defined in detail? Were the tools or methods used to measure exposure accurate and reliable–for example, have they been validated or are they objective? This issue is important as it influences confidence in the reported exposures. When exposures are measured with less accuracy or validity, it is

harder to see an association between exposure and outcome even if one exists. Also as important is whether the exposures were assessed in the same manner within groups and between groups; if not, bias may result.

For example, retrospective self-report of dietary salt intake is not as valid and reliable as prospectively using a standardized dietary log plus testing participants' urine for sodium content. Another example is measurement of BP, where there may be quite a difference between usual care, where clinicians measure BP however it is done in their practice setting (which can vary considerably), and use of trained BP assessors using standardized equipment (e.g., the same BP device which has been tested and calibrated) and a standardized protocol (e.g., patient is seated for 5 minutes with feet flat on the floor, BP is taken twice in each arm, and all four measurements are averaged). In each of these cases, the former would get a "no" and the latter a "yes."

Here is a final example that illustrates the point about why it is important to assess exposures consistently across all groups: If people with higher BP (exposed cohort) are seen by their providers more frequently than those without elevated BP (nonexposed group), it also increases the chances of detecting and documenting changes in health outcomes, including CVD-related events. Therefore, it may lead to the conclusion that higher BP leads to more CVD events. This may be true, but it could also be due to the fact that the subjects with higher BP were seen more often; thus, more CVD-related events were detected and documented simply because they had more encounters with the health care system. Thus, it could bias the results and lead to an erroneous conclusion.

Question 10. Repeated exposure assessment

Was the exposure for each person measured more than once during the course of the study period? Multiple measurements with the same result increase our confidence that the exposure status was correctly classified. Also, multiple measurements enable investigators to look at changes in exposure over time, for example, people who ate high dietary sodium throughout the followup period, compared to those who started out high then reduced their intake, compared to those who ate low sodium throughout. Once again, this may not be applicable in all cases. In many older studies, exposure was measured only at baseline. However, multiple exposure measurements do result in a stronger study design.

Question 11. Outcome measures

Were the outcomes defined in detail? Were the tools or methods for measuring outcomes accurate and reliable–for example, have they been validated or are they objective? This issue is important because it influences confidence in the validity of study results. Also important is whether the outcomes were assessed in the same manner within groups and between groups.

An example of an outcome measure that is objective, accurate, and reliable is death–the outcome measured with more accuracy than any other. But even with a measure as objective as death, there can be differences in the accuracy and reliability of how death was assessed by the investigators. Did they base it on an autopsy report, death certificate, death registry, or report from a family member? Another example is a study of whether dietary fat intake is related to blood cholesterol level (cholesterol level being the outcome), and the cholesterol level is measured from fasting blood samples that are all sent to the same laboratory. These examples would get a "yes." An example of a "no" would be self-report by subjects that they had a heart attack, or self-report of how much they weigh (if body weight is the outcome of interest).

Similar to the example in Question 9, results may be biased if one group (e.g., people with high BP) is seen more frequently than another group (people with normal BP) because more frequent encounters with the health care system increases the chances of outcomes being detected and documented.

Question 12. Blinding of outcome assessors

Blinding means that outcome assessors did not know whether the participant was exposed or unexposed. It is also sometimes called "masking." The objective is to look for evidence in the article that the person(s) assessing the outcome(s) for the study (for example, examining medical records to determine the outcomes that occurred in the exposed and comparison groups) is masked to the exposure status of the participant. Sometimes the person measuring the exposure is the same person conducting the outcome assessment. In this case, the outcome assessor would most likely not be blinded to exposure status because they also took measurements of exposures. If so, make a note of that in the comments section.

As you assess this criterion, think about whether it is likely that the person(s) doing the outcome assessment would know (or be able to figure out) the exposure status of the study participants. If the answer is no, then blinding is adequate. An example of adequate blinding of the outcome assessors is to create a separate committee, whose members were not involved in the care of the patient and had no information about the study participants' exposure status. The committee would then be provided with copies of participants' medical records, which had been stripped of any potential exposure information or personally identifiable information. The committee would then review the records for prespecified outcomes according to the study protocol. If blinding was not possible, which is sometimes the case, mark "NA" and explain the potential for bias.

Question 13. Followup rate

Higher overall followup rates are always better than lower followup rates, even though higher rates are expected in shorter studies, whereas lower overall followup rates are often seen in studies of longer duration. Usually, an acceptable overall followup rate is considered 80 percent or more of participants whose exposures were measured at baseline. However, this is just a general guideline. For example, a 6-month cohort study examining the relationship between dietary sodium intake and BP level may have over 90 percent followup, but a 20-year cohort study examining effects of sodium intake on stroke may have only a 65 percent followup rate.

Question 14. Statistical analyses

Were key potential confounding variables measured and adjusted for, such as by statistical adjustment for baseline differences? Logistic regression or other regression methods are often used to account for the influence of variables not of interest.

This is a key issue in cohort studies, because statistical analyses need to control for potential confounders, in contrast to an RCT, where the randomization process controls for potential confounders. All key factors that may be associated both with the exposure of interest and the outcome–that are not of interest to the research question–should be controlled for in the analyses.

For example, in a study of the relationship between cardiorespiratory fitness and CVD events (heart attacks and strokes), the study should control for age, BP, blood cholesterol, and body weight, because all of these factors are associated both with low fitness and with CVD events. Well-done cohort studies control for multiple potential confounders.

Some general guidance for determining the overall quality rating of observational cohort and cross-sectional studies

The questions on the form are designed to help you focus on the key concepts for evaluating the internal validity of a study. They are not intended to create a list that you simply tally up to arrive at a summary judgment of quality.

Internal validity for cohort studies is the extent to which the results reported in the study can truly be attributed to the exposure being evaluated and not to flaws in the design or conduct of the study–in other words, the ability of the study to draw associative conclusions about the effects of the exposures being studied on outcomes. Any such flaws can increase the risk of bias.

Critical appraisal involves considering the risk of potential for selection bias, information bias, measurement bias, or confounding (the mixture of exposures that one cannot tease out from each other). Examples of confounding include co-interventions, differences at baseline in patient characteristics, and other issues throughout the questions above. High risk of bias translates to a rating of poor quality. Low risk of bias translates to a rating of good quality. (Thus, the greater the risk of bias, the lower the quality rating of the study.)

In addition, the more attention in the study design to issues that can help determine whether there is a causal relationship between the exposure and outcome, the higher quality the study. These include exposures occurring prior to outcomes, evaluation of a dose-response gradient, accuracy of measurement of both exposure and outcome, sufficient timeframe to see an effect, and appropriate control for confounding–all concepts reflected in the tool.

Generally, when you evaluate a study, you will not see a "fatal flaw," but you will find some risk of bias. By focusing on the concepts underlying the questions in the quality assessment tool, you should ask yourself about the potential for bias in the study you are critically appraising. For any box where you check "no" you should ask, "What is the potential risk of bias resulting from this flaw in study design or execution?" That is, does this factor cause you to doubt the results that are reported in the study or doubt the ability of the study to accurately assess an association between exposure and outcome?

The best approach is to think about the questions in the tool and how each one tells you something about the potential for bias in a study. The more you familiarize yourself with the key concepts, the more comfortable you will be with critical appraisal. Examples of studies rated good, fair, and poor are useful, but each study must be assessed on its own based on the details that are reported and consideration of the concepts for minimizing bias.

Last Updated March 2014

Supplemental Material S4(b). Manual quality assessment of included studies using the National Institute of Health Quality Assessment Tool for Observational Cohort and Cross-Sectional Studies (National Institute of Health, 2021).

**Study**	**Q1**	**Q2**	**Q3**	**Q4**	**Q5**	**Q6**	**Q7**	**Q8**	**Q9**	**Q10**	**Q11**	**Q12**	**Q13**	**Q14**	**Total Score**	**Overall Quality Rating**
Jhamb and Singh (2022)	✓	✓	✓	✓	✗	✗	✗	✗	✗	*NA*	✗	✗	*NA*	✗	4/12	Poor
Horsley et al. (2020)	✓	✓	*NR*	✓	✗	✗	✗	✗	✓	*NA*	✓	*NA*	*NA*	✗	5/11	Fair
Rejeb et al. (2017)	✓	✓	✓	✓	✗	✓	✓	✓	✓	✗	✓	✗	✓	✓	11/13	Good
Sankaravel et al. (2017)	✓	✓	✓	✓	✗	✗	✗	✗	✗	*NA*	✗	✗	*NA*	✗	4/12	Poor
Talabi et al. (2014)	✓	✓	*NR*	✓	✗	✗	✗	✗	✗	*NA*	✗	✗	*NA*	✗	3/11	Poor
Okhovatian and Ezatolahi (2009)	✓	✓	✓	✓	✗	✗	✗	✓	✓	*NA*	✗	✗	*NA*	✗	6/12	Poor
Meyer et al. (2007)	✓	✓	✓	✓	✓	✗	✗	✓	✓	*NA*	✓	✗	*NA*	✓	9/12	Good
Persic et al. (2006)	✓	✓	✓	✓	✗	✗	✗	✓	✓	*NA*	✗	✗	*NA*	✓	7/12	Fair
Parkkari et al. (2004)	✓	✓	✓	✓	✗	✓	✓	✓	✓	✗	✓	✗	*NA*	✓	10/12	Good
Eime et al. (2003)	✓	✓	*NR*	✓	✗	✗	✗	✗	✓	*NA*	✓	*NA*	*NA*	✗	5/11	Fair
Chard and Lachmann (1987)	✓	✗	*NR*	✗	✗	✗	✗	✓	✗	*NA*	✓	✗	*NA*	✗	3/12	Fair
Berson et al. (1981)	✓	✓	✓	✗	✗	✗	✗	✓	✗	*NA*	✗	✗	*NA*	✗	4/12	Poor

Abbreviations: NA, not applicable; NR, not reported.

Supplemental Material S5. Study Characteristics.

Source	Number of Participants	Setting and Location	Age Range of Participants	Methodology
Jhamb and Singh (2022)	120 squash players (selected from a total of 134 responses)	Various squash clubs based in India	Mean 41 years	Retrospective descriptive survey using an online questionnaire (via Google Suite)
Horsley et al. (2020)	67 elite squash players (45 males, 22 females)	Those funded by England Squash, UK	18-35 (mean 25 years)	Retrospective analysis of injury records from 2004 to 2015 using modified Orchard Sports Injury Classification System (OSICS)
Rejeb et al. (2017)	18 male squash players (out of a total of 166 athletes aged 12-18)	Middle Eastern Youth Sports Academy	12-18 years old	Prospective injury surveillance using medical records
Sankaravel et al. (2017)	60 squash players (out of 94 approached)	Sukma Games 2016, Kuching, Sarawak, Malaysia	Mean: 17.6 years	Retrospective survey using the Standardised Nordic Questionnaire (SNQ)
Talabi et al. (2013)	187 squash players	48 squash clubs across 25 states, Nigeria	20-60 years	Cross-sectional survey using structured questionnaire
Okhovatian and Ezatolahi (2009)	52 squash players	Squash Federation in Enghelab Sport Complex (SFESC), Iran	Mixed (mean ~30s)	Questionnaire-based interviews on injury history from September 2006 to September 2008
Meyer et al. (2007)	106 adolescent squash players	Three high schools in Western Cape high school squash league, South Africa	13-18 years	Retrospective descriptive survey (recall period of four weeks) using a structured self-administered questionnaire
Persic et al. (2006)	653 individuals<br>600 squash players	Evenly across Switzerland, Germany, and France	Mixed (juniors to professionals)	Cross-sectional survey using a predefined questionnaire regarding orofacial injuries
Parkkari et al. (2004)	3363 randomly selected participants, aged 15-74 (92% of 3657 recruited)	Finland (Nationwide)	15-74 years	Prospective cohort study using three structured telephone interviews spread over one year
Eime et al. (2003)	389 squash injury cases (50 hospital admissions, 339 ED presentations)	Various hospitals in Victoria, Australia	Not specified	Retrospective analysis of hospital admissions and emergency department data
Chard and Lachmann (1987)	372 squash players (out of a total of 631 racquet sport players)	Sports Injury Clinic, Addenbrooke's Hospital, Cambridge, UK	Adolescents to adults (59% over 25 years)	Eight-year retrospective study analysing racquet sport-related injuries within clinic records
Berson et al. (1981)	200 squash players (155 responses)<br>100 squash players (83 responses)<br>100 squash players (72 responses)	Private squash club, New York City, USA<br>Public squash club, New York City, USA	Majority over 40 years	Retrospective telephone survey using structured interviews to collect injury history

Supplemental Material S6. Key Findings and Risk Factors.

Source	Details of Injury or Illness	Risk Factors
Jhamb and Singh (2022)	• 86% had injury requiring more than two weeks for recovery.<br>• Contact injury = 1.92% (N=2/104).<br>• Lower limb injuries = 67.3%.<br>• Lower back = 31.73%.<br>• Upper limb = 19.23%.<br>• Eye = 0.96%.<br>• Most common: knee (21%), back (11%), elbow (10%) …<br>• Average time to recovery = 5.8 months; ACL injuries take the longest (9.25 months).	• Poor technique.<br>• Inadequate physical conditioning.<br>• Level of caution applied.<br>• Slightly older population (>34 years).<br>• Poor conditioning and technique.<br>• Incomplete rest between injuries.
Horsley et al. (2020)	• Lower limb (mostly soft tissue) = 76.48% of all injuries.<br>• Ankle/heel (20.81%), thigh (12.69%), knee (10.83%), hip/groin (9.48%), buttock (7.45%), lower leg (7.61%), foot (7.61%).<br>• Injury rate: 0.46 injuries per athlete per month.	• High intensity and training load.<br>• Aged 18–23 (due to physiological changes related to muscle strength, flexibility, and co-ordination).<br>• Previous injury (leading to reduced joint range of motion or strength resulting in possible injury).
Rejeb et al. (2017)	• Time-loss = 87% of all injuries.<br>• Overall injury incidence = 5.5 (95% CI: 5.1–6.0) per 1000 hours of exposure.<br>• Incidence of time-loss injuries = 4.8 (95% CI: 4.4–5.2) per 1000 hours of exposure.<br>• Incidence of growth conditions = 1.2 (95% CI: 1.0–1.4) per 1000 hours of exposure.<br>• Incidence of serious injuries = 0.6 (95% CI: 0.5–0.8) per 1000 hours of exposure.<br>• Prevalence of overuse injuries = 50.3% .<br>• Growth conditions = 20.0%.<br>• Most of the injuries (67.0%) affected the lower limb; foot and ankle were most commonly injured (22.0%).<br>• Knee injuries mostly followed joint overuse, whereas foot and ankle injuries were often attributed to an acute cause.<br>• Minor and moderate injuries accounted for 87.0% of injuries.<br>• Muscle, tendon, and osteochondrosis injuries accounted for 52.0% of injuries.<br>• Squash had the highest injury incidence, at 8.5 injuries per athlete.<br>• Higher levels of exposure were associated with a greater overuse relative risk (RR = 1.03, 95% CI: 1.01–1.014, $p < 0.001$).<br>• 51.9% of injuries resulted in an absence of less than seven days.	• High exposure and overuse.<br>• Reduced motor coordination and proprioceptive skills.<br>• High-level sport was demanded from adolescents who were actively growing and maturing.
Sankaravel et al. (2017)	• 83.33% had MSK symptoms.<br>• Most common: wrist/hand (31.7%), ankle/feet (26.7%), shoulder (25%), hip/thigh and knee (20%), neck and upper back (16.7%), lower back (11.7%), elbow (8.3%).<br>• Not specified; chronic overuse likely.	• Increased playing frequency.<br>• Longer play history.<br>• Unsuitable equipment.

Talabi et al. (2012)	• Types: soft tissue (85.70%), sprain (10.30%), fracture (2.70%), other (1.30%).<br>• Severity: acute (55.2%), chronic (26.6%), over-use (18.2%).<br>• Causes: self-inflicted (40.1%), opponent-assisted (29.9%), opponent-caused (19.3%), other (10.7%).	• Self-inflicted (40.1%).<br>• Improper positional play.<br>• Poor fitness and agility.<br>• Poor floor conditions.<br>• Poor footwear.
Okhovatian and Ezatolahi (2009)	• Most common injuries: lower back pain (36.5%), tennis elbow (21%), general muscle strain (19%), racket kicking (19%), ball kicking (19%), hamstring strain (12%), and knee sprain (10%).	• Inappropriate eye protection.<br>• Poor technique.<br>• Increased training load.<br>• Club-level players.
Meyer et al. (2007)	• 29% injured in prior 4 weeks (N=31).<br>• Most common regions: thigh (19%), lower back (13%), and shoulder (13%).<br>• The most common mechanism was 'pain without a traumatic incident'.<br>• 53% minor, 34% moderate, 13% serious.	• High levels of strain on adolescent players, soft-tissue inflexibility.<br>• Insufficient warm-up.<br>• Lack of experience.<br>• Inadequate coaching and supervision of younger players.<br>• Pre-existing injury.
Persic et al. (2006)	• Most common tooth injuries were crown fractures, avulsion, and dislocation.	• Poor awareness regarding management of an avulsed tooth<br>• Low mouthguard uptake (N=1/600).
Parkkari et al. (2004)	• Squash carried highest injury rate within study (18.3/1000 hrs).<br>• Level I = 71%; Level II = 24%; Level III = 6%.	• High intensity (competitions).<br>• Adolescent injuries are most frequent concerning recreational and competitive sports.
Eime et al. (2003)	• Lower extremities are most frequently injured (sprain or strain) at 34.7 injuries per 100,000 players.<br>• Overall, the injury rate of eye injuries was 19.0 per 100,000 players; eye injuries were the most common ED presentation.<br>• 72% of hospital admissions stayed for less than two days; all others stayed between two and seven days.<br>• After ED presentations, only 6.2% were admitted to the hospital.	• Common causes of injury (highest to lowest): overexertion or strenuous movements, struck or knocked (not further specified), unintentional (not further specified), hit or crushed, struck by object, collision with person, fall, other.<br>• Risk factors: shoe-surface interaction, fitness, skill level, age, and gender of participants.
Chard and Lachmann (1987)	• Most common injuries: knee, lumbar region, ankle, muscles (especially calf).<br>• "Muscle injuries were not more commonly seen in older players, but no assessment of severity could be made."<br>• "The injuries seen in this study were not insignificant, as the majority required physiotherapy, and some of those with more severe injuries, especially in the knee, needed referral to the orthopaedic department."	• Older players (high level of physical stress less tolerated by body tissues).<br>• Inadequate warm-up.<br>• New players.<br>• Infrequent play.
Berson et al. (1981)	• 44.5% injury rate (N=69/155).<br>• Most common injuries were strains, sprains, fractures, and lacerations.<br>• 47% of injuries considered disabling (out of action for more than two weeks); players over 40 affected for longer.	• Lack of warm-up, fatigue, poor body condition, previous injury.<br>• Unsuitable footwear, court conditions.<br>• Aged 40 and above.<br>• Increased playing frequency (females).

Supplemental Material S7. Reported Distribution of Squash Injuries.

Supplemental Material S7(a). Distribution of Squash Injuries by Anatomical Region.

Study	Number of Injuries Reported Within the Study					
	**Head and neck**	**Upper limb**	**Trunk**	**Lower limb**	**Unspecified**	**Multiple regions**
Jhamb and Singh (2022)	-	25	3	76	-	-
Horsley et al. (2020)	11	27	117	437	-	-
Sankaravel et al. (2017)	10	39	17	40	-	-
Rejeb et al. (2017)	Data not provided					
Talabi et al. (2012)	28	30	-	119	10	-
Okhovatian and Ezatolahi (2009)	3	13	25	20	28	-
Meyer et al. (2007)	-	17	9	22	-	-
Persic et al. (2006)	253	-	-	-	-	-
Parkkari et al. (2004)	Data not provided					
Eime et al. (2003)	173	67	9	113	27	-
Chard and Lachmann (1987)	-	80	77	215	-	-
Berson et al. (1981)	-	16	7	33	13	-
Total Per Region	478	314	264	1075	78	-
Total Overall	2209					
Confidence Interval (CI)*	95% CI = 0.2164 ± 0.0172	95% CI = 0.1421 ± 0.0146	95% CI = 0.1195 ± 0.0135	95% CI = 0.4866 ± 0.0208	95% CI = 0.0353 ± 0.0077	-
Proportion Per Region	478/2209 = 21.64%	314/2209 = 14.21%	264/2209 = 11.95%	1075/2209 = 48.66%	78/2209 = 3.53%	-
Finalised Proportion Per Region	95% CI: (19.92%, 23.36%)	95% CI: (12.76%, 15.67%)	95% CI: (10.60%, 13.30%)	95% CI: (46.58%, 50.75%)	95% CI: (2.76%, 4.30%)	-

*CIs calculated via Agresti-Coull method ($CI = \hat{p} \pm Z_{\alpha/2}\sqrt{\frac{\hat{p}(1-\hat{p})}{n}}$), where $\hat{p}$ is the sample proportion (x/n), $Z_{\alpha/2}$ is the Z-score (1.96), and n is the total sample size (2209).

Supplemental Material S7(b). Distribution of Squash Injuries by Tissue Type.

Source	Muscle / Tendon	Nervous	Bone	Cartilage / Synovium / Bursa	Ligament / Joint capsule	Superficial tissues / skin	Vessels	Stump	Internal organs	Non-specific
Jhamb and Singh (2022)	73	-	4	3	24	-	-	-	-	-
Horsley et al. (2020)	418	6	46	18	104	-	-	-	-	-
Sankaravel et al. (2017)	Data not provided									
Rejeb et al. (2017)	Data not provided									
Talabi et al. (2012)	160	-	5	-	18	-	-	-	-	3
Okhovatian and Ezatolahi (2009)	47	-	3	3	13	-	-	-	-	23
Meyer et al. (2007)	27	-	-	4	3	-	-	-	-	14
Persic et al. (2006)	-	-	27	-	-	184	-	-	60 (eye)	-
Parkkari et al. (2004)	Data not provided									
Eime et al. (2003)	Data not provided									
Chard and Lachmann (1987)	Data not provided									
Berson et al. (1981)	18	-	2	13	11	25	-	-	-	-
Total Per Region	743	6	87	41	173	209	-	-	60	40
Total Overall	1359									
Confidence Interval (CI)*	95% CI = 0.5467 ± 0.0265	95% CI = 0.0044 ± 0.0035	95% CI = 0.0640 ± 0.0130	95% CI = 0.0302 ± 0.0091	95% CI = 0.1273 ± 0.0177	95% CI = 0.1538 ± 0.0192	95% CI = 0.00 ± 0.0022	95% CI = 0.00 ± 0.0022	95% CI = 0.0442 ± 0.0109	95% CI = 0.0294 ± 0.0090
Proportion Per Region	743/1359 = 54.67%	6/1359 = 0.44%	87/1359 = 6.40%	41/1359 = 3.02%	173/1359 = 12.73%	209/1359 = 15.38%	0/1359= 0.00%	0/1359= 0.00%	60/1359 = 4.42%	40/1359 = 2.94%
Finalised Proportion Per Region	95% CI: (52.02%, 57.32%)	95% CI: (0.09%, 0.79%)	95% CI: (5.10%, 7.70%)	95% CI: (2.11%, 3.93%)	95% CI: (10.96%, 14.50%)	95% CI: (13.46%, 17.30%)	95% CI: (0%, 0.22%)	95% CI: (0%, 0.22%)	95% CI: (3.33%, 5.51%)	95% CI: (2.04%, 3.84%)

*CIs calculated via Agresti-Coull method ($CI = \hat{p} \pm Z_{\alpha/2}\sqrt{\frac{\hat{p}(1-\hat{p})}{n}}$), where $\hat{p}$ is the sample proportion (x/n), $Z_{\alpha/2}$ is the Z-score (1.96), and n is the total sample size (2209).

Supplemental Material S8. Categorisation of Reported Lower Limb Injuries by Anatomical Region.

**Lower Extremity Region**	**Specific Structure/Injury**	**Study Reference**
Knee	Cruciate Ligament Injuries	Jhamb and Singh (2022), Chard and Lachmann (1987)
	Medial Meniscus Injury	Jhamb and Singh (2022), Chard and Lachmann (1987)
	Chronic Pain (Knee)	Jhamb and Singh (2022), Rejeb et al. (2017)
	Knee Sprain	Horsley et al. (2020), Okhovatian and Ezatolahi (2009)
	Collateral Ligament Injuries	Chard and Lachmann (1987)
	Traumatic Synovitis	Chard and Lachmann (1987)
	Patellar Injuries	Chard and Lachmann (1987)
Ankle/Foot/Heel	Ankle Sprains	Chard and Lachmann (1987), Berson et al. (1981)
	Foot And Ankle Injuries (Combined)	Rejeb et al. (2017), Sankaravel et al. (2017)
	Achilles Tendon Injuries	Jhamb and Singh (2022), Chard and Lachmann (1987)
	Heel Pain	Jhamb and Singh (2022)
	Bunion	Jhamb and Singh (2022)
	Toe Fracture	Jhamb and Singh (2022)
Thigh	Thigh Musculature	Horsley et al. (2020), Rejeb et al. (2017), Meyer et al. (2007)
	Hamstring Strain	Jhamb and Singh (2022), Okhovatian and Ezatolahi (2009)
	Quadriceps Strain and Tear	Jhamb and Singh (2022)
Hip/Groin/Buttock	Hip/Groin	Horsley et al. (2020), Rejeb et al. (2017)
	Groin Strain	Jhamb and Singh (2022)
	Hip Pain	Jhamb and Singh (2022)
	Buttock	Horsley et al. (2020)
Lower Leg (excluding ankle/foot)	Calf Muscle Tear	Jhamb and Singh (2022)
	Gastrocnemius Strain	Jhamb and Singh (2022)
	Shin Splints	Jhamb and Singh (2022)

Supplemental Material S9. Certainty of Evidence Assessment.

Supplemental Material S9(a). GRADE Assessment Table.

Key Finding	No. of Studies	Risk of Bias	Inconsistency	Indirectness	Imprecision	Publication Bias	Overall Certainty
The lower extremity is the most frequently injured region in squash.	10	Serious[1]	Not Serious[2]	Not Serious[3]	Serious[4]	Serious[5]	⊕⊕○○ Low
Ankles and knees are the most common sites of lower extremity injuries.	10	Serious[1]	Serious[6]	Not Serious[3]	Serious[4]	Serious[5]	⊕⊕○○ Low
Soft tissue injuries are the most common injury type overall.	8	Serious[1]	Not Serious[7]	Not Serious[3]	Serious[4]	Serious[5]	⊕⊕○○ Low
Overuse injuries carry a substantial risk within squash.	6	Serious[1]	Not Serious[8]	Not Serious[3]	Serious[4]	Serious[5]	⊕⊕○○ Low
Acute injuries are commonly caused by striking (ball/racket), falls, or collisions.	8	Serious[1]	Serious[9]	Not Serious[3]	Serious[4]	Serious[5]	⊕○○○ Very Low
Age, gender, skill level, and previous injury may influence a player's personal injury risk.	7	Serious[1]	Very Serious[10]	Not Serious[3]	Very Serious[11]	Serious[5]	⊕○○○ Very Low

Supplemental Material S9(b). Downgrading Justification.

1. Most studies utilised retrospective data, which significantly increased the chance of recall bias and selective outcome reporting.
2. Most findings note the lower limb as a predominant injury site, ranging from 32% to 76.48% of all injuries.
3. The literature comprised a wide demographical range of squash players, increasing applicability to the broader population.
4. Reported injury rates varied from 0.45 to 18.3 injuries per 1000 hours; this significant variation makes it more challenging to determine a true injury rate.
5. Only published literature was used within this review, which could have resulted in over-representation of positive findings.
6. High heterogeneity across studies makes it difficult to confidently determine the most common primary injury site.
7. Among studies which reported injuries as per tissue type, soft tissue was most consistently reported.
8. Among studies which discussed acute and chronic injury patterns, overuse injuries were frequently recognised.
9. Strikes, falls, and collisions are commonly reported, but there is clear variation regarding their frequency and overall significance.

10. Certain risk factors appear contradictory: some studies suggest that younger players presented with an overall higher injury rate, but some other studies suggest that players over 40 carry the higher risk; some findings imply that beginners and amateurs have a higher injury risk, whereas others state that more experienced players can sustain more serious and/or more frequent injuries; some studies report males as more commonly affected by injury proportionally; however, other studies suggest that females carry a higher risk alongside increased playing time.
11. The literature does not provide specific effect estimates for age, gender, or skill level, such as relative risks or odds ratios. Instead, it generally describes the risks qualitatively, without provision of precise data, which can limit the accuracy that is often provided through epidemiological calculations.

Supplemental Material S10. Completed PRISMA Checklists for Reporting of Systematic Reviews.

Supplemental Material S10(a). PRISMA 2020 Checklist.

Topic	No.	Item	Location where item is reported
**TITLE**			
**Title**	1	Identify the report as a systematic review.	Title Page
**ABSTRACT**			
**Abstract**	2	See the PRISMA 2020 for Abstracts checklist	-
**INTRODUCTION**			
**Rationale**	3	Describe the rationale for the review in the context of existing knowledge.	Introduction (Section 1.4)
**Objectives**	4	Provide an explicit statement of the objective(s) or question(s) the review addresses.	Introduction (Section 1.5)
**METHODS**			
**Eligibility criteria**	5	Specify the inclusion and exclusion criteria for the review and how studies were grouped for the syntheses.	Methods (Section 2.1, 2.2)
**Information sources**	6	Specify all databases, registers, websites, organisations, reference lists and other sources searched or consulted to identify studies. Specify the date when each source was last searched or consulted.	Methods (Section 2.2) & Supplementary Material 2
**Search strategy**	7	Present the full search strategies for all databases, registers and websites, including any filters and limits used.	Supplementary Material 2
**Selection process**	8	Specify the methods used to decide whether a study met the inclusion criteria of the review, including how many reviewers screened each record and each report retrieved, whether they worked independently, and if applicable, details of automation tools used in the process.	Methods (Section 2.2)
**Data collection process**	9	Specify the methods used to collect data from reports, including how many reviewers collected data from each report, whether they worked independently, any processes for obtaining or confirming data from study investigators, and if applicable, details of automation tools used in the process.	Methods (Section 2.2)
**Data items**	10a	List and define all outcomes for which data were sought. Specify whether all results that were compatible with each outcome domain in each study were sought (e.g. for all measures, time points, analyses), and if not, the methods used to decide which results to collect.	Methods (Section 2.2) & Supplementary Material 3
	10b	List and define all other variables for which data were sought (e.g. participant and intervention characteristics, funding sources).	Methods (Section 2.2) & Supplementary Material 3

		Describe any assumptions made about any missing or unclear information.	
**Study risk of bias assessment**	11	Specify the methods used to assess risk of bias in the included studies, including details of the tool(s) used, how many reviewers assessed each study and whether they worked independently, and if applicable, details of automation tools used in the process.	Methods (Section 2.3)
**Effect measures**	12	Specify for each outcome the effect measure(s) (e.g. risk ratio, mean difference) used in the synthesis or presentation of results.	Methods (Section 2.5)
**Synthesis methods**	13a	Describe the processes used to decide which studies were eligible for each synthesis (e.g. tabulating the study intervention characteristics and comparing against the planned groups for each synthesis (item 5)).	Methods (Section 2.5)
	13b	Describe any methods required to prepare the data for presentation or synthesis, such as handling of missing summary statistics, or data conversions.	Methods (Section 2.5)
	13c	Describe any methods used to tabulate or visually display results of individual studies and syntheses.	Results (Section 3.1, 3.4), Tables 1 & 2, Figure 2
	13d	Describe any methods used to synthesize results and provide a rationale for the choice(s). If meta-analysis was performed, describe the model(s), method(s) to identify the presence and extent of statistical heterogeneity, and software package(s) used.	Methods (Section 2.5)
	13e	Describe any methods used to explore possible causes of heterogeneity among study results (e.g. subgroup analysis, meta-regression).	Results (Section 3.4), Discussion (Section 4.3)
	13f	Describe any sensitivity analyses conducted to assess robustness of the synthesized results.	Methods (Section 2.5)
**Reporting bias assessment**	14	Describe any methods used to assess risk of bias due to missing results in a synthesis (arising from reporting biases).	Methods (Section 2.5), Discussion (Section 4.7)
**Certainty assessment**	15	Describe any methods used to assess certainty (or confidence) in the body of evidence for an outcome.	Methods (Section 2.4), Supplementary Material 9
**RESULTS**			
**Study selection**	16a	Describe the results of the search and selection process, from the number of records identified in the search to the number of studies included in the review, ideally using a flow diagram.	Results (Section 3.1), Figure 1
	16b	Cite studies that might appear to meet the inclusion criteria, but which were excluded, and explain why they were excluded.	Figure 1
**Study characteristics**	17	Cite each included study and present its characteristics.	Results (Section 3.1), Table 1,

			Supplementary Material 5
**Risk of bias in studies**	18	Present assessments of risk of bias for each included study.	Supplementary Material 4
**Results of individual studies**	19	For all outcomes, present, for each study: (a) summary statistics for each group (where appropriate) and (b) an effect estimate and its precision (e.g. confidence/credible interval), ideally using structured tables or plots.	Results (Sections 3.2, 3.3), Tables 1 & 2, Supplementary Material 6
**Results of syntheses**	20a	For each synthesis, briefly summarise the characteristics and risk of bias among contributing studies.	Results (Section 3.4)
	20b	Present results of all statistical syntheses conducted. If meta-analysis was done, present for each the summary estimate and its precision (e.g. confidence/credible interval) and measures of statistical heterogeneity. If comparing groups, describe the direction of the effect.	Results (Section 3.4), Figure 2
	20c	Present results of all investigations of possible causes of heterogeneity among study results.	Results (Section 3.4), Discussion (Section 4.3)
	20d	Present results of all sensitivity analyses conducted to assess the robustness of the synthesized results.	Results (Section 3.4)
**Reporting biases**	21	Present assessments of risk of bias due to missing results (arising from reporting biases) for each synthesis assessed.	Discussion (Section 4.7)
**Certainty of evidence**	22	Present assessments of certainty (or confidence) in the body of evidence for each outcome assessed.	Discussion (Section 4.5), Supplementary Material 9
**DISCUSSION**			
**Discussion**	23a	Provide a general interpretation of the results in the context of other evidence.	Discussion (Sections 4.1-4.6)
	23b	Discuss any limitations of the evidence included in the review.	Discussion (Section 4.3)
	23c	Discuss any limitations of the review processes used.	Discussion (Section 4.7)
	23d	Discuss implications of the results for practice, policy, and future research.	Discussion (Sections 4.8)
**OTHER INFORMATION**			
**Registration and protocol**	24a	Provide registration information for the review, including register name and registration number, or state that the review was not registered.	Methods (Section 2.1)
	24b	Indicate where the review protocol can be accessed, or state that a protocol was not prepared.	Methods (Section 2.1), Supplementary Material 1

	24c	Describe and explain any amendments to information provided at registration or in the protocol.	Methods (Section 2.1)
**Support**	25	Describe sources of financial or non-financial support for the review, and the role of the funders or sponsors in the review.	Funding Statement (Not applicable)
**Competing interests**	26	Declare any competing interests of review authors.	Conflicts of Interest Statement (No conflicts of interest)
**Availability of data, code and other materials**	27	Report which of the following are publicly available and where they can be found: template data collection forms; data extracted from included studies; data used for all analyses; analytic code; any other materials used in the review.	Data Availability Statement, Supplementary Material 3

Supplemental Material S10(b). PRISMA 2020 for Abstracts Checklist.

Topic	No.	Item	Reported?
**TITLE**			
**Title**	1	Identify the report as a systematic review.	Yes
**BACKGROUND**			
**Objectives**	2	Provide an explicit statement of the main objective(s) or question(s) the review addresses.	Yes
**METHODS**			
**Eligibility criteria**	3	Specify the inclusion and exclusion criteria for the review.	No
**Information sources**	4	Specify the information sources (e.g. databases, registers) used to identify studies and the date when each was last searched.	No
**Risk of bias**	5	Specify the methods used to assess risk of bias in the included studies.	Yes
**Synthesis of results**	6	Specify the methods used to present and synthesize results.	Yes
**RESULTS**			
**Included studies**	7	Give the total number of included studies and participants and summarise relevant characteristics of studies.	No
**Synthesis of results**	8	Present results for main outcomes, preferably indicating the number of included studies and participants for each. If meta-analysis was done, report the summary estimate and confidence/credible interval. If comparing groups, indicate the direction of the effect (i.e. which group is favoured).	Yes
**DISCUSSION**			
**Limitations of evidence**	9	Provide a brief summary of the limitations of the evidence included in the review (e.g. study risk of bias, inconsistency and imprecision).	Yes
**Interpretation**	10	Provide a general interpretation of the results and important implications.	Yes
**OTHER**			
**Funding**	11	Specify the primary source of funding for the review.	No
**Registration**	12	Provide the register name and registration number.	No
